# Systemic DPP4 activity is reduced during primary HIV‐1 infection and is associated with intestinal RORC ^+^ CD4^+^ cell levels: a surrogate marker candidate of HIV‐induced intestinal damage

**DOI:** 10.1002/jia2.25144

**Published:** 2018-07-10

**Authors:** Mickaël J Ploquin, Armanda Casrouge, Yoann Madec, Nicolas Noël, Beatrice Jacquelin, Nicolas Huot, Darragh Duffy, Simon P Jochems, Luca Micci, Camille Lécuroux, Faroudy Boufassa, Thijs Booiman, Thalia Garcia‐Tellez, Mathilde Ghislain, Roger Le Grand, Olivier Lambotte, Neeltje Kootstra, Laurence Meyer, Cecile Goujard, Mirko Paiardini, Matthew L Albert, Michaela Müller‐Trutwin

**Affiliations:** ^1^ Institut Pasteur Unité HIV Inflammation et Persistance Paris France; ^2^ Institut Pasteur Unité Immunobiologie des cellules dendritiques Paris France; ^3^ INSERM U1223 Paris France; ^4^ Institut Pasteur URE Epidémiologie des Maladies Emergentes Paris France; ^5^ Assistance Publique – Hôpitaux de Paris Service de Médecine Interne et Immunologie Clinique Groupe Hospitalier Universitaire Paris Sud, Hôpital Bicêtre Le Kremlin‐Bicêtre France; ^6^ IDMIT Department CEA Université Paris Sud Inserm U1184 Immunology of viral infections and auto‐immune diseases (IMVA) IBFJ Fontenay‐aux‐Roses and Kremlin‐Bicêtre France; ^7^ Université Paris Sud Le Kremlin Bicêtre France; ^8^ Emory University School of Medicine and Yerkes National Primate Research Center Atlanta Georgia USA; ^9^ INSERM CESP U1018 Université Paris Sud Le Kremlin‐Bicêtre France; ^10^ Academisch Medisch Centrum Laboratory of Viral Immune Pathogenesis Amsterdam The Netherlands; ^11^ Present address: Liverpool School of Tropical Medicine Liverpool UK; ^12^ Present address: Department of Cancer Immunology Genentech Inc. San Francisco CA USA

**Keywords:** HIV, SIV, inflammation, intestine, dipeptidylpeptidase, biomarker, Th17

## Abstract

**Introduction:**

Combined anti‐retroviral therapy (cART) transformed HIV‐1 from a deadly disease into a chronic infection, but does not cure HIV infection. It also does not fully restore HIV‐induced gut damage unless administered extremely early after infection. Additional biomarkers are needed to evaluate the capacity of therapies aimed at HIV remission/cure to restore HIV‐induced intestinal immune damage and limit chronic inflammation. Herein, we aimed to identify a systemic surrogate marker whose levels would reflect gut immune damage such as intestinal Th17 cell loss starting from primary HIV‐1 infection.

**Methods:**

Biomarker discovery approaches were performed in four independent cohorts, covering HIV‐1 primary and chronic infection in 496 naïve or cART‐treated patients (Amsterdam cohort (ACS), ANRS PRIMO, COPANA and CODEX cohorts). The concentration and activity of soluble Dipeptidylpeptidase 4 (sDPP4) were quantified in the blood from these patients, including pre‐ and post‐infection samples in the ACS cohort. For quantification of DPP4 in the gut, we utilized two non‐human primate models, representing pathogenic (macaque) and non‐pathogenic (African green monkey) SIV infection. Four gut compartments were analysed in each animal model (ileum, jejunum, colon and rectum) for quantification of *DPP4*,*RORC* and *TBX21* gene expression in sorted CD4^+^ cells. To analyse if sDPP4 levels increase when Th17 cells were restored, we quantified sDPP4 in plasma from SIV‐infected macaques treated with IL‐21.

**Results:**

We showed that sDPP4 levels were strongly decreased in primary HIV‐1 infection. Strikingly, sDPP4 levels in primary HIV‐1 infection predicted time to AIDS. They were not increased by cART in chronic HIV‐1 infection (median 36 months on cART). In the gut of SIV‐infected non‐human primates, *DPP4* mRNA was higher in CD4^+^ than CD4^−^ leucocytes. DPP4 specifically correlated with RORC expression, a Th17 marker, in CD4^+^ cells from the intestine. We further demonstrated that sDPP4 activity levels were increased in animals treated with IL‐21 and that this increase was associated with restoration of the Th17 compartment and reduced inflammation. Furthermore, *DPP4* mRNA levels in small intestine CD4^+^ cells positively correlated with circulating DPP4 activity.

**Conclusion:**

These data provide evidence that blood sDPP4 levels could be useful as a correlate for HIV‐induced intestinal damage.

## 1 Introduction

Combined anti‐retroviral treatment (cART) drastically improves life expectancy and quality of life of HIV‐infected individuals 1, 2 but does not result in viral cure. Despite sustained undetectable viraemia during efficient cART, the virus persists in so‐called viral reservoirs 3, 4, 5. In addition, a residual chronic inflammation persists in treated patients 6, 7, 8, 9. Indeed, while cART lowers chronic inflammation concomitantly with a reduction in viral load, in most patients the inflammation levels are not normalized and remain higher than in healthy persons 6, 8. Even in spontaneous HIV controllers (HIC), the inflammation level remains most often higher as compared to healthy donors and those HIC with higher inflammatory scores have a higher risk for CD4^+^ T‐cell loss and disease progression 10, 11. Of note, the residual and persistent inflammation under effective cART is associated with a higher risk of comorbidities and non‐AIDS illnesses, such as cancer and cardiovascular diseases 12, 13.

A therapy leading to complete viral eradication 3, 14, or with higher likelihood to long‐term undetectable viraemia in the absence of cART 15, is the aspirational goal. This ideal scenario would allow treatment cessation without an increased risk of transmission to partners, while also reducing inflammation and fully restoring immune function and tissue integrity, reducing the risk of increased comorbidities and non‐AIDS mortality.

In the absence of cART, HIV‐1 infection induces a rapid depletion of CD4^+^ T cells in the gut, in particular Th17 cells, and epithelial barrier disruption ensues along with associated microbial translocation contributing to chronic inflammation 16, 17, 18. It has been shown that cART initiation at the earliest stages of HIV‐1 infection (Fiebig stages I and II), prevents Th17 cell depletion and fully reverses the initially observed mucosal and systemic immune activation 19, 20. By contrast, cART initiation at Fiebig stage III restored Th17 cell numbers, but not their polyfunctionality, and patients maintained elevated mucosal and systemic CD8^+^ T‐cell activation post initiation of cART 19. In the majority of the cases, cART is initiated during chronic HIV‐1 infection. It generally results in good immune reconstitution in peripheral blood, however, reconstitution in the gut occurs at a much slower pace, where both immunological and structural abnormalities persist even in long‐term treated patients 21. Of particular interest are therapeutic approaches aimed at reversing intestinal damage 22, 23, 24, 25, 26, 27. It is unclear so far if one of these agents could be useful, although a few pilot studies provided some promising results. Treatment with mesalamine, which is used in clinics to decrease mucosal inflammation in ulcerative colitis, did not change the persistent T‐cell activation in rectum nor plasma sCD14, IL‐6, D‐dimer levels during HIV‐infection 22. Rifaximin, a non‐absorbable antibiotic that decreases LPS in cirrhotics, had a marginal impact on microbial translocation in patients on cART 25. Treatment with the drug Sevelamer, which binds microbial lipopolysaccharide in the gut, seems to confer cardiovascular benefits but its capacity to block microbial translocation is unclear 24. In several studies, probiotic therapy or therapy with probiotics supplemented with IL‐21 showed promising effects, such as enhanced CD4+ T‐cell restoration in the gut and/or decrease in inflammation and microbial translocation 28, 29, 30. IL‐21 alone resulted in transient increases of rectal Th17 cells and decrease in systemic inflammation 31. To guide drug development in this area, systemic biomarkers evaluating the restoration of gut integrity and immune function are urgently required. Several systemic markers of gut epithelial barrier disruption are currently used in the field of HIV 18, 32, 33, 34. These markers include I‐FABP, sCD14, LBP, LPS and bacterial RNA, the latter two being a direct measurement of microbial translocation 35. While the use of these markers has helped the field to make incredible progress in the understanding of HIV physiopathology, few are entirely specific for microbial translocation or epithelial disruption in the gut (particularly sCD14, related to monocyte activation) and some are difficult to measure. Also, none of these biomarkers serve as a surrogate for intestinal Th17 cell levels in the gut.

Th17 cells have been described to express the highest levels of Dipeptidylpeptidase 4 (DPP4) among CD4^+^ T cells 36. DPP4 is a membrane‐associated enzyme (also known as CD26), which can exist in soluble form in blood. It cleaves hormones and chemokines such as Glucagon‐like peptide, SDF‐1, all three CXCR3‐ligands (IP‐10, MIG, I‐TAC), RANTES, CCL11 and CCL20 37. It was previously reported to be increased in hepatitis C (HCV) infection, acting to inhibit trafficking of CXCR3^+^ leucocytes to the sites of viral replication and possibly accounting for viral immune escape 38. In contrast to HCV, a loss of circulating DPP4^+^CD4^+^ T cells and reduced plasma soluble DPP4 (sDPP4) activity has been observed during chronic HIV infection 39, 40, 41, 42, 43. Here, we examined whether sDPP4 levels in the blood can be used as a systemic marker of intestinal Th17 levels and gut immune damage during HIV infection.

## 2 Methods

### 2.1 Ethics statement

Patient enrolment respected all European guidelines and those established by the World Medical Association in its declaration of Helsinki. All patients were adult subjects and gave their written informed consent. The scientific board of the Amsterdam cohort studies (ACS) approved this study. Concerning the French ANRS cohorts, the Paris‐Cochin Ethics Committee approved the study protocols for the patients from PRIMO C06 and COPANA C09 cohorts, and the Comité de Protection des Personnes Ile‐de‐France VII, Paris, France (approval reference: 05–22) approved the CODEX CO21 study protocol. The Institut Pasteur “Comité de recherché Clinique” (CoRC #2013‐05) equally approved this study.

Animals were housed in the facilities of the CEA (“Commissariat à l'Energie Atomique”, Fontenay‐aux‐Roses, France) IDMIT facilities (Infrastructure for Infectious Disease Models and Innovative Therapies), Fontenay‐aux‐Roses, France (permit number A 92‐032‐02) or the Institut Pasteur, Paris, France (permit number A 78‐100‐3). All experimental procedures were conducted in strict accordance with the European guideline 2010/63/UE on the protection of animals used for scientific purposes (French decree 2013‐118) and with the recommendations of the Weatherall report. The monitoring of the animals was under the supervision of the veterinarians in charge of the animal facilities. All efforts were made to minimize suffering, including efforts to improve housing conditions and to provide enrichment opportunities in the IDMIT Center (e.g., 12∶12 light dark schedule, provision of monkey biscuits supplemented with fresh fruits and constant water access, objects to manipulate, interaction with caregivers and research staff). All procedures were performed under anaesthesia using 10 mg of ketamine per kg body weight. Euthanasia was performed prior to the development of any symptoms of disease. Euthanasia was done by IV injection of a lethal dose of pentobarbital. The CEA is in compliance with Standards for Human Care and Use of Laboratory of the Office for Laboratory Animal Welfare (OLAW, USA) under OLAW Assurance number #A5826‐01. Animal experimental protocols were approved by the Ethical Committee of Animal Experimentation (CETEA‐DSV, IDF, France; Notification number: 10‐051b).

The analyses of the human and animal study samples took place between 2013 and 2016.

### 2.2 Patient cohorts and samples

A large serum library derived from cART‐naïve patients, including pre‐infection samples from patients enrolled in the Amsterdam Cohort Studies (ACS) of HIV infection and AIDS was used (Table S1, 44). None of the 136 subjects studied here had started anti‐retroviral therapy when samples were collected. These patients had an estimated date of seroconversion (SC) defined as the midpoint between the date of the last visit with a negative HIV test and the first visit with a positive HIV test (complete or incomplete western blot) 45. Samples collected before infection were obtained between 24 and 3 months before the estimated date of SC. Patients co‐infected with other bloodborne pathogens (HIV‐2, HBV, HCV) were excluded.

The viremic cART‐naive subjects (VIR, n = 121) were part of the French ANRS C09 COPANA cohort (Table S1) 44, 46. A subgroup from the COPANA Cohort of 40 patients received cART (>24 months of follow‐up and >12 months with VL < 50 copies/mL) 47. In addition, 31 randomly selected cART‐treated patients were analysed. The median time on cART was 36 months (IQR, 27 to 66 months).

The HIC (n = 82) were enrolled in the French ANRS C021 CODEX cohort (Table S1, 44). To be enrolled, patients had to be diagnosed as HIV‐infected for more than five years and to remain cART‐naive with viraemia below 400 copies/mL in at least five consecutive assays, regardless of their CD4 cell count 10.

The 126 subjects enrolled in the French ANRS C06 PRIMO cohort were previously described 48. Primary HIV infection (PHI) (M0) was defined by an incomplete WB, with detectable HIV RNA load and/or P24 protein. Most patients in PHI were in Fiebig stage III/IV. Patients were categorized as rapid progressors (RP), slow progressors (SP) or normal progressors (P) as previously described 48. Briefly, RP were defined as those losing their peripheral CD4+ T cells to below 350 cells/mm^3^ after PHI in <12 months; P showed more than 350 CD4^+^ T cells/mm^3^ after 12 months but <500 cells/mm^3^ at least at one time point before or at 42 months post‐PHI, in the absence of treatment; and SP were displaying more than 500 CD4+ T cells/mm^3^ after 42 months post‐PHI in the absence of treatment. The cell‐associated viral DNA load has been extensively studied in these patients 49, 50, 51, 52.

None of the participants in the ANRS cohorts who we studied received immunosuppressive drugs, IFN therapy or chemotherapy, and none had cancer, autoimmune diseases or HIV‐unrelated chronic inflammatory metabolic disorders at enrolment.

Frozen plasma from blood collected on EDTA was obtained from the ANRS cohorts.

EDTA plasma from HIV/HBV/HCV‐seronegative individuals (Healthy Donors (HD), n = 87) were obtained from *Etablissement Français du Sang* (EFS, Paris, France) for research purposes.

As mentioned above, in the ACS cohort, the patients with HCV infection were excluded. In the ANRS PRIMO and COPANA cohorts, only two individuals per cohort were HCV infected. In the 82 patients from the Codex cohort, 19 were HCV infected (23%).

### 2.3 Non‐human primates

Five Chinese rhesus macaques (*Macaca mulatta*) were I.V. infected with 50 AID_50_ (animal infectious dose 50) of an uncloned SIVmac251 isolate (provided by A. M. Aubertin, Université Louis Pasteur, Strasbourg, France). Twelve Indian rhesus macaques were I.V. infected with 300 TCID_50_ (tissue culture infectious dose 50). SIVmac_239_ as previously reported 31. Five African green monkeys (*Chlorocebus sabaeus,* AGM) were I.V. infected with 250 TCID_50_ of purified SIVagm.sab92018 wild‐type isolate 53, 54, 55. Plasma was obtained from blood collected on EDTA. Intestinal samples from the AGM and the Chinese rhesus macaques were collected at sacrifice and treated as described below in the following paragraph. The longitudinal collection and treatment of the rectal samples from the Indian rhesus macaques have been previously reported 53, 54, 55.

### 2.4 Isolation of CD4^+^ and CD4^−^ cells from simian gut tissue

At necropsy (day 65 post‐infection), a fragment (5/7 cm in length) was collected from sections of the intestine (jejunum, ileum, colon and rectum) of all five Chinese rhesus macaques and five AGM, except for the ileum fragment that was collected in 3/5 macaques and 3/5 AGMs. The fragments were enzymatically dissociated in RPMI culture medium (Life Technologies, Carlsbad, CA, USA) containing collagenase (Collagenase II‐S, Sigma‐Aldrich, Lyon, France) and DNAse (Sigma‐Aldrich) for 1 hour with agitation (80 rpm) at 37°C. Total leucocytes were separated from epithelial/endothelial cells through a Percoll gradient. CD4‐positive leucocytes were purified with magnetic beads (Miltenyi columns and CD4 cell purification kit) and the CD4‐negative fraction collected.

### 2.5 DPP4 assays

The concentrations of sDPP4 were measured with the commercial sandwich ELISA (human DPP4/DPP4 Duo Set ELISA, R&D Systems) following the supplier's instructions, as described 38. Briefly, Maxisorp plates (Nunc) were coated with 2 μg/mL of the commercial capture antibody in PBS overnight at room temperature. The wells were saturated with 300 μL of 1% in PBS for 1 hour. A seven‐point standard curve was performed with the highest concentration at 2000 pg/mL. Plasma samples were used at 1/800 dilution. Plates were analysed in a Labsystems Multiskan MS (Thermo) device.

The sDPP4 enzymatic activity was measured using a luciferase‐based assay (DPP4‐Glo™ Protease Assay; Promega). This assay utilizes a luminogenic DPP substrate, Gly‐Pro‐aminoluciferin. After cleavage of the proximal two amino acids from the substrate, the aminoluciferin is free to engage luciferase. Relative luminescent units (RLU) are proportional to the DPP activity. This assay was performed on plasma samples serially diluted between 0.025%, 0.25% and 2.5% in 10 mm Tris–HCL pH 8 with 0.1% Prionex stabilizer (Calbiochem). The enzymatic activity was measured following the manufacturer's instructions, as previously described 38, 56, 57. Briefly, in a white plate (Greiner bio‐one), 50 μL of kit reagent containing the luciferase substrate was added to 50 μL of sample. Maximal signal is reached within 30 minutes and is stable for 3 hours. Recombinant human DPP4 (Sigma D4943) was used as a reference. A seven‐point standard curve, using twofold serial dilutions and duplicates of each dilution was applied. DPP activity was expressed in units/ml based on supplier information after subtraction of the background signal (PBS or medium only). Supplier unit definition was: one unit will produce 1.0 micromole of P‐Nitroaniline from Gly‐Pro‐P‐Nitroanilide per minute at pH 7.6 at 37°C. Plates were read in a Tristar LB941 device (Berthold Technologies, Oak Ridge, TN, USA). To account for potential effects on enzymatic activity in HIV infection, activity values were normalized by concentration, and both types of results are presented below.

### 2.6 Quantification of IP‐10

The quantification of IP‐10 (also known as CXCL10) was previously reported 44, 48 and based on enzyme‐linked immunosorbent assays. Briefly, the IP‐10 concentrations were determined in stored plasma or sera samples (−80°C) using the commercially available Quantikine CXCL10 assay (R&D Systems, Minneapolis, Minnesota) according to the manufacturers’ instruction.

### 2.7 Quantification of gene expression

Total RNA was extracted and reverse‐transcribed as previously described 58. qPCR (Taqman chemistry) and commercial kits were used to quantify the expression levels of genes of interest in the simian samples as previously reported 44. Briefly, the Taqman primers and probes used were Hs00175210 for *DPP4*, Hs01076112_m1 for *RORc*, Hs00203436_m1 for *Tbx21* and 4310893^E^ for 18S. The expression of each gene was normalized to that of 18S rRNA, and relative expression levels were calculated using the ΔΔ*CT* method using as the internal reference, the raw value for each gene in rectal CD4+ leucocytes from one rhesus macaque or, alternatively, by normalizing each value against the raw value of each gene in CD4+ leucocytes enriched from the rectum of each animal, as previously described 44.

The probes were initially designed for human sequences. We aligned the sequences that contain the primers and probes to the sequences from AGM and MAC (Figure S1). The *DPP4* sequence was 100% identical between human, AGM and MAC. The Tbx21 sequences of AGM and MAC were 97.6% identical to the human sequence and 100% identical to each other. The RORc sequences were 100% identical in the region that covers the probe between the three species and 96.8% to 98.4% identical in the remaining part. For the 18S sequence, the corresponding region is not available for AGM in the databases. The 18S MAC sequence was identical to the human one except for a four‐base pair insertion outside the region that covers the probe region. For 18S, we previously showed that the values obtained for AGM and MAC by RT‐qPCR using 18SrRNA for normalization correlated with the values obtained by a distinct quantification method (microarrays) 59, indicating that the use of 18S rRNA for quantification of AGM and MAC mRNA gives robust results. Moreover, we can exclude that differences in the levels of *DPP4* and *Tbx21* mRNA as measured here are due to sequence differences. For *RORc* it is potentially possible but unlikely.

### 2.8 Statistical analyses

Continuous variables from the human cohorts were reported as medians and 25th to 75th percentiles (interquartile range, IQR). They were compared across groups with a Kruskall–Wallis test followed by Dunn's test for multiple group comparisons, or a student *t*‐test or Wilcoxon non‐parametric tests for two‐group comparisons, depending on the sample size. Categorical variables were compared across groups using the chi‐squared test. Logistic regression was used to identify factors associated with rapid progression (RP, <350 CD4/mm^3^ by M12 post‐seroconversion). In the ACS, time‐to‐event analyses were used to analyse AIDS‐related mortality and progression to CD4 levels <200 cells/mm^3^. For both outcomes, time‐to‐event was described using Kaplan–Meier non‐parametric estimates and compared using the log‐rank test. The Cox proportional hazard model was used to identify factors associated with the outcome. Correlations between markers were evaluated by calculating the Pearson correlation coefficient or Spearman's correlation coefficient, depending on the normality of the data distribution and the group size. A *p* < 0.05 was considered statistically significant. All analyses were performed with Stata 13 software (StataCorp, College Station, Texas, USA) or GraphPad PRISM 6 (GraphPad software, La Jolla CA, USA). Plots and graphs were designed with GraphPad PRISM 6 (GraphPad software).

## 3 Results

### 3.1 Soluble plasma DPP4 is decreased in primary HIV infection

**Figure 1:**
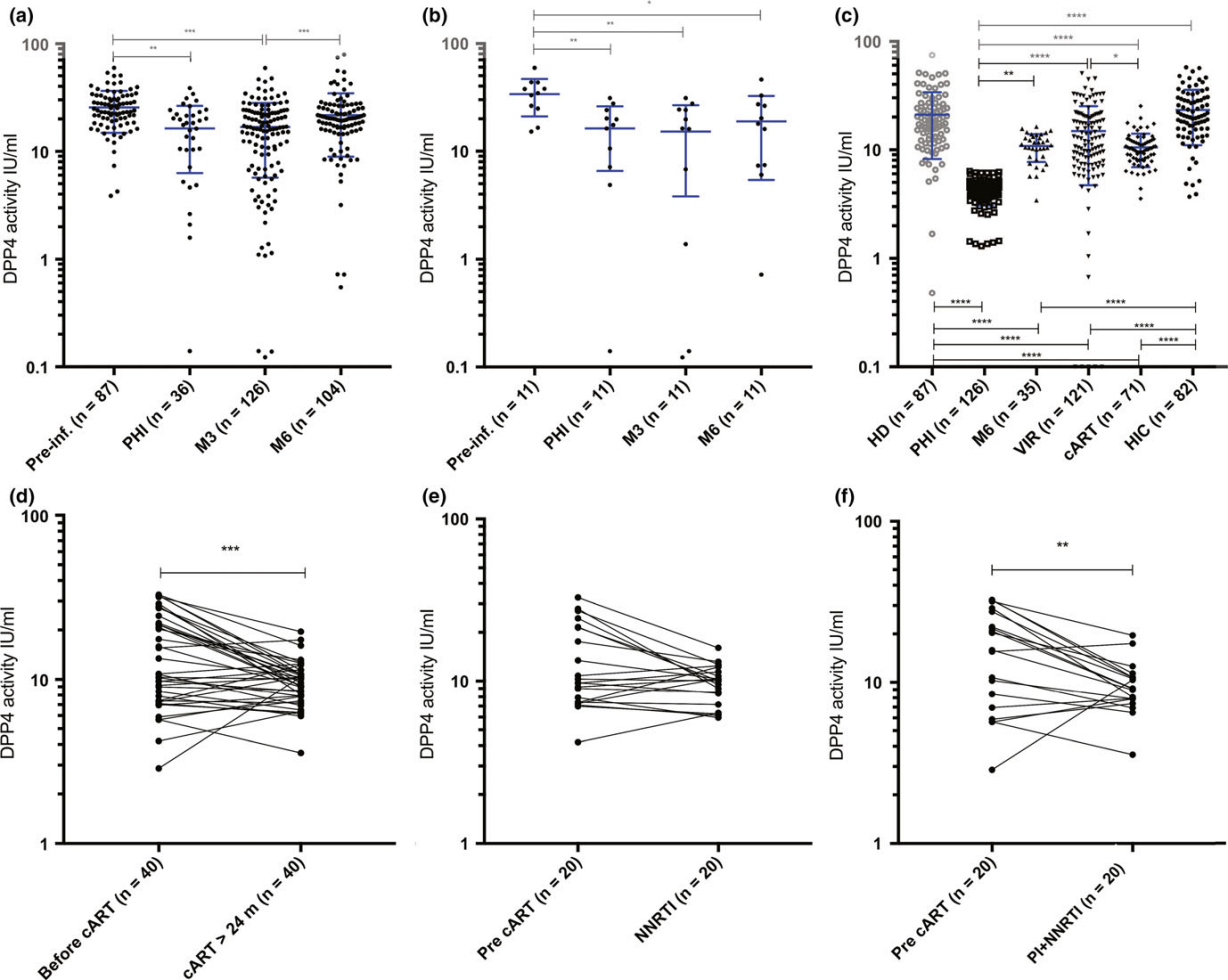
Soluble DPP4 levels in blood over time during distinct phases of HIV‐1 infection. **(a)** Soluble DPP4 activity levels over time in patients from the Amsterdam cohort Study (ACS). Blood collected before HIV‐1 infection and at early time points post‐infection were analysed. (**b)**. Soluble DPP4 activity levels in 11 of these patients from the ACS for whom samples at all four time points of the study were available. **(c)** Soluble DPP4 activity levels in healthy donors (HD), in HIV‐infected treatment‐naive patients at early time points of infection (primary infection, PHI and six months post‐PHI) from the ANRS CO6 cohort, in the chronic phase of infection (viremic patients (VIR)) from the ANRS COPANA cohort, and during controlled infection, either cART‐treated patients (cART) from the COPANA cohort and HIV controllers (HIC) from the ANRS CODEX cohort. **(d‐f)** Evolution of sDPP4 levels before and during cART in 40 patients from the ANRS COPANA cohort. The values from the same patient are connected through a line to show the individual evolution of the sDPP4 levels before and after cART initiation. **(d)** Pre‐ and post‐ART sDPP4 activity (UI/mL) in the 40 cART‐treated patients. Twenty of these patients had received Protease inhibitors (PI). **(e)** Pre‐ and post‐ART sDPP4 activity (UI/mL) in 20 NRTI‐based cART‐treated patients. **(f)** Pre‐ and post‐ART sDPP4 activity (UI/mL) in the 20 patients on cART with PI‐containing regimen. Pre‐inf. = before HIV‐1 infection; PHI =  primary HIV‐1 infection; M3 = three months post‐seroconversion; M6 = 6 months post‐seroconversion (panel **a‐b** in the ACS) or post‐PHI (panel C in the PRIMO cohort). For graphs **a**,** b** and **c**, the median +interquartile range are shown. For graphs **a** and **c**, the student *t*‐test was used. For graphs **b**,** d**,** e** and **f**, the Wilcoxon sign‐rank test for paired data was used. *p < 0.05; **p < 0.01; ***p < 0.001; ****p < 0.0001.

It has been shown that circulating sDPP4 is reduced during chronic HIV infection 43. If peripheral sDPP4 is a potential marker of intestinal Th17 loss, DPP4 loss would be rapid and already decreased during primary HIV‐1 infection (PHI). We thus measured sDPP4 in the Amsterdam HIV Cohort (ACS) (Table S1). The ACS disposes of a large collection of stored samples from individuals before and after HIV‐1 infection. This cohort therefore enabled evaluation of sDPP4 levels in acute infection compared with the pre‐infection period to document changes in response to HIV infection. In total, 136 patients were studied. sDPP4 was quantified before infection (N = 87), in primary HIV infection (PHI, N = 36), at three months post‐seroconversion (M3, N = 126) and six months post‐seroconversion (M6, N = 104). In most studies, the levels of sDDP4 are measured at the level of its enzymatic activity and absolute concentrations are more rarely presented 39, 56, 60, 61, 62. We measured both and as in previous studies 57, found a strong correlation between the concentration and enzymatic activity of sDDP4 (*R* = 0.72, *p* < 0.0001 and Figure S2A). We thus performed the subsequent analyses with regard to the DPP4 activity levels. The levels of sDPP4 activity were statistically significantly lower during PHI and at M3 when compared to pre‐infection (Figure 1a). Individuals for whom samples were available at all four time points (N = 11), showed a similar profile (Figures 1b, S2B). At M6, the sDPP4 activity was higher than at M3, but in some cases still lower than pre‐infection (Figure 1b, S2B).

We then examined if such a decrease in sDPP4 in PHI could also be observed in an independent cohort by quantifying sDPP4 in patients from the French ANRS PRIMO cohort 51. The plasma samples of blood from 126 patients recruited in PHI and 87 healthy donors were analysed. sDPP4 was statistically significantly lower in PHI when compared to healthy donors (Figure 1c). The sDPP4 levels in PHI were lower than in the ACS cohort (Figure 1a), but it cannot be excluded that this may be due to an earlier Fiebig stage in patients from the PRIMO cohort.

These data demonstrate that the levels of sDPP4 were already decreased in PHI. Then sDPP4 activity recovered progressively, but remained lowered compared to healthy donors.

### 3.2 Soluble DPP4 levels were not restored under cART

To study whether sDPP4 activity could be restored by cART, we quantified sDPP4 before and during cART in patients from the ANRS COPANA cohort, who were enrolled less than six months after diagnosis of HIV infection (Table S1). The levels of sDPP4 in cART patients (N = 71) were compared to those of viremic patients before cART (N = 122), HIC (N = 82) and healthy donors (N = 87). Patients were on cART for >24 months, with effective viral control for >12 months (<50 copies of HIV RNA/mL).

The sDPP4 activity levels in the HIC were similar to those in healthy donors, but higher than in all other HIV groups, including the cART‐treated individuals (Figure 1c). Among the 82 HIC, 19 were HCV infected. A high activity of sDPP4 was previously reported in HCV infection 38. We compared the sDPP4 levels in the 19 HCV‐infected HIC to those from the 63 HCV‐negative HIC and observed no difference (sDPP4 IU/mL : *p* = 0.18; sDPP4 ng/mL : *p* = 0.34; sDPP4 IU/ng : *p* = 0.55; Wilcoxon non‐parametric test). Furthermore, when we removed the 19 patients with HCV infection and analysed the 63 remaining HCV‐negative HIV controllers, the sDDP4 levels in the HIC were still higher than in all other HIV groups, while not statistically significantly different from those in healthy donors (Table S2). Hence, we did not observe any impact of the HCV infection on the sDPP4 levels in the HIC studied here, and when comparisons were performed considering only HIC who are HCV‐negative, the sDPP4 levels in the HIC were still higher than in all other HIV groups.

The sDPP4 activity levels in the viremic patients and cART patients were lower than those observed in healthy donors. Surprisingly, sDPP4 activity levels did not increase after cART (Figure 1c). In order to further analyse this observation, we compared sDPP4 levels in 40 patients on cART for whom we also had a blood sample just before cART initiation. By comparing the same patients longitudinally, we observed a decrease in sDPP4 after cART initiation (Figures 1d, S2C). The median fold change in sDPP4 before and after cART initiation was 0.77. Since cART regimens often comprise a protease inhibitor and because it is unknown whether the inhibitor can have an impact on the enzymatic activity of DPP4, we analysed separately the individuals receiving a cART regimen with or without protease inhibitor. The decrease in sDPP4 remained statistically significant when considering the 20 patients who benefited from a combination of drugs containing a Protease inhibitor (median fold change 0.62), but not in patients who did not receive any PI (median fold change 0.86) as part of their therapeutic regimen (Figures 1e,f, S2D,E). Other parameters such as duration of infection were not different between the two groups.

The levels of sDPP4 levels were therefore not restored under cART and in some cases, even further decreased.

### 3.3 Low‐soluble DPP4 in primary HIV infection predicts rapid progression

**Figure 2:**
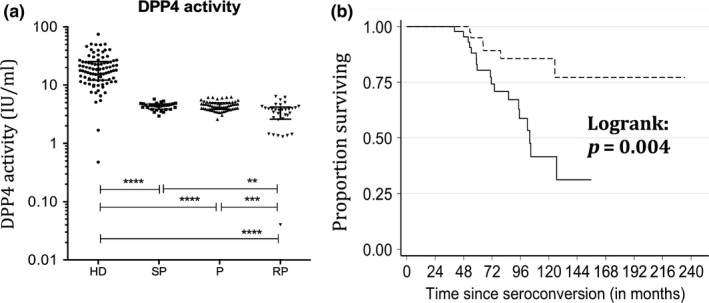
Soluble blood DPP4 levels with regard to disease progression profiles. Soluble DPP4 levels were quantified during primary HIV‐1 infection in patients from the ANRS PRIMO cohort displaying distinct profiles of disease progression (SP, slow progressors; P, normal progressors; RP, rapid progressors; HD, healthy donors). **(a)** sDPP4 enzymatic activity per ml of blood. The median levels were 18.0 for HD, 4.4 for SP and P and 3.8 for RP. **(b)** Kaplan–Meier survival analysis of AIDS‐related death by sDPP4 levels measured six months after seroconversion (M6) (≤ or > to the median) in treatment‐naïve patients from the Amsterdam cohort. The dotted line corresponds to sDPP4 levels > median and the solid line to sDDP4 levels < median at M6. For graph **a,** the Wilcoxon non‐parametric test was used and for graph **b** the log‐rank test. The median and interquartile range is shown in graph **a**. **p < 0.01; ***p < 0.001; ****p < 0.0001.

We next analysed if the levels of sDPP4 during the early phase of the infection were associated with the disease progression profile. For this, the 126 patients from the PRIMO cohort were stratified into three groups, according to the kinetics of CD4^+^ T‐cell loss (slow progressors, typical progressors, rapid progressors), as described previously 48 and in the methods section. The enzymatic activity of sDPP4 in PHI was reduced most strongly in those patients who became rapid progressors, that is, reached CD4^+^ T‐cell counts <350 within one year after PHI (Figures 2a, S3).

Logistic regression analyses confirmed that patients with low levels of sDPP4 activity display an increased risk of becoming a rapid disease progressor (Table 1). We then analysed sDPP4 compared to other markers predictive of disease progression, that is, viraemia, CD4^+^ T‐cell counts and IP‐10. The latter has been identified as an early biomarker of rapid progression 48. Multivariate analyses showed that sDPP4 was an independent marker of disease progression when including CD4 T‐cell levels, HIV RNA or DNA viral loads (Table 1). Of note, low levels of sDPP4 activity in PHI were better associated with rapid disease progression than viraemia or cell‐associated HIV DNA levels, although less than IP‐10 (“co factor values,” Table 1).

**Table 1:** Logistic regression for evaluation of the capacity of sDPP4 in PHI compared with other markers to predict disease progression

	sDPP4	Adj. OR (95% CI)	*p*	Co‐factor	Adj. OR (95% CI)	*p*
Absolute level of sDPP4 activity (IU/mL)	≤4.18 >4.18	4.56 (1.72 to 12.12) 1	**0.002**	IP10 ≤233 >233	1 9.78 (3.32 to 28.75)	**<0.001**
	≤4.18 >4.18	4.98 (1.81 to 13.71) 1	**0.002**	CD4, cells/mm^3^* ≤350 351 to 500 501 to 750 >750	7.45 (1.14 to 48.54) 0.74 (0.26 to 2.10) 1 0.22 (0.05 to 1.09)	**0.026**
	≤4.18 >4.18	4.32 (1.66 to 11.23) 1	**0.003**	CD4, cells/mm^3^* ≤500 501 to 750 >750	1.19 (0.47 to 2.98) 1 0.22 (0.05 to 1.07)	0.12
	≤4.18 >4.18	4.13 (1.64 to 10.38) 1	**0.003**	vRNA, log cp/mL ≤4.00 4.01 to 5.00 >5.00	1 6.67 (0.75 to 56.95) 9.30 (1.13 to 76.76)	0.11
	≤4.18 >4.18	3.80 (1.51 to 9.59) 1	**0.005**	ca‐DNA, log cp/10^6^ PBMC** ≤2.85 >2.85	1 2.37 (0.72 to 7.78)	0.16
Normalized level of sDPP4 activity (IU/ng)	≤0.010 >0.010	3.79 (1.48 to 9.71) 1	**0.006**	IP10 ≤233 >233	1 10.27 (3.5 to 30.1)	**<0.001**
	≤0.010 >0.010	2.66 (1.08 to 6.53) 1	**0.033**	CD4, cells/mm^3^* ≤350 351 to 500 501 to 750 >750	4.41 (0.75 to 26.07) 0.81 (0.30 to 2.24) 1 0.19 (0.04 to 0.89)	**0.043**
	≤0.010 >0.010	2.93 (1.21‐7.08) 1	**0.017**	CD4, cells/mm^3^* ≤500 501 to 750 >750	1.15 (0.46 to 2.87) 1 0.19 (0.04 to 0.89)	0.078
	≤0.010 >0.010	3.54 (1.48 to 8.52) 1	**0.005**	vRNA, log cp/mL ≤4.00 4.01 to 5.00 >5.00	1 6.64 (0.79 to 56.23) 11.43 (1.4 to 93.98)	0.055
	≤0.010 >0.010	2.87 (1.20 to 6.87) 1	**0.018**	ca‐DNA, log cp/10^6^ PBMC** ≤2.85 >2.85	1 2.95 (0.92 to 9.46)	0.069

Logistic regression was performed to evaluate the capacity of DPP4 compared to other markers to predict disease progression by bivariate analysis. We analysed both the absolute levels of sDPP4 enzymatic activity as well as the levels of normalized sDPP4 activity. The analyses were performed on data from the 126 patients of the PRIMO cohort. The viral load and IP‐10 levels were determined in 48. If fewer samples were available, this is indicated in the table: *125 patients, **117 patients. All values were from PHI. The thresholds for DPP4 and IP10 were based on the median; for the CD4 count they correspond to historical thresholds for anti‐retroviral treatment initiation (350 and 500 cells/mm^3^) and an arbitrary threshold at 750 cells/mm^3^ was also used as the distribution was off‐centred towards larger values. For plasma viral RNA, we used thresholds at 4 and 5 log copies/mL as these are cutoffs usually used in the literature in pathogenesis studies to separate progressors from long‐term non‐progressors 80; for DNA, we used 25th percentile to gain statistical power as the univariate analysis in four categories showed an effect below the threshold. vRNA, plasma HIV RNA; ca‐DNA, cell (PBMC)‐associated viral DNA; Adj. OR, adjusted odds ratio; CI, confidence interval; cp, copies. Values of *p* < 0.05 are indicated in bold.

Lastly, survival analyses were performed in the patients who were followed until clinical symptoms of AIDS and death (Amsterdam cohort). sDPP4 levels at M6, but not viraemia, predicted the time until AIDS‐related death (*p* = 0.004) (Figure 2b).

In summary, we show that sDPP4 levels rapidly decrease during PHI, are not fully restored by cART initiated during chronic infection and predict disease progression.

### 3.4 DPP4 expression profiles in the gut resemble those of RORC in intestinal CD4+ cells during SIV infection

**Figure 3:**
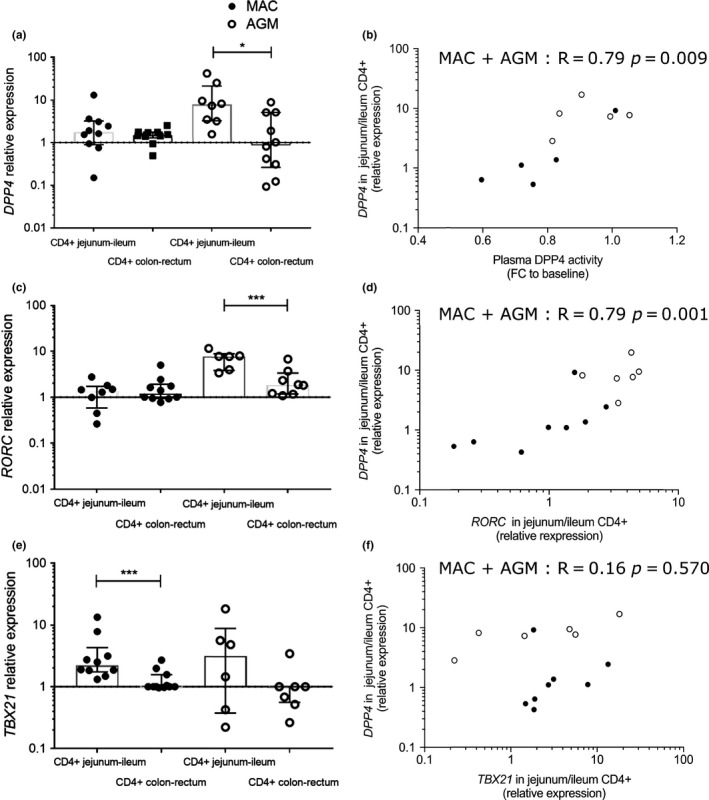
*DPP4* mRNA levels in the gut in pathogenic and non‐pathogenic SIV infection. (**a,c,e)** Four intestinal compartments (ileum, jejunum, colon, rectum) from five rhesus macaques and five AGM were studied at an early phase of infection (day 65 pi.). CD4+ leucocytes were enriched from the distinct sections of the intestine. The values for the small intestine (ileum, jejunum) and large intestine (rectum, colon) were pooled. For MAC, the available material was: jejunum, colon and rectum for five animals, ileum for three to five animals; For AGM, the available samples were: jejunum, colon and rectum for four to five animals, ileum for four to five animals). When the material was limited, we privileged the analyses of DPP4mRNA. **(a)** *DPP4* mRNA levels in intestinal CD4^+^ cells. **(b)** *DPP4* mRNA expression in CD4^+^ cells from the small intestine (jejunum) from the five MAC and five AGM plotted against plasma sDPP4 activity in blood from the same animals. **(c)** *RORC* mRNA levels in intestinal CD4^+^ cells. **(d**) *RORC* mRNA expression levels plotted against *DPP4* mRNA in CD4^+^ cells from the small intestine. **(e)** *TBX21* (*t‐bet*) mRNA levels in intestinal CD4^+^ cells. **(f)** *TBX21* mRNA expression levels plotted against *DPP4* mRNA in CD4^+^ cells from the small intestine. The correlations were statistically significant when the two species were pooled, but not within individual species. **(b,d,f**) Each circle represents the value of one tissue sample. Black (full circles): MAC; white (open circles): AGM. For graphs **a**,** c** and **e** the Wilcoxon non‐parametric test was used; for graphs **b**,** d** and **f** the Spearman non‐parametric correlation was used. The median and interquartile range are shown in graphs **a**,** c** and **e**. FC, fold change. *p <  0.05; **p < 0.01; ***p < 0.001.

Since DPP4 has been described to be expressed at high levels in the gut 36, we next examined whether the reduction in these biomarker levels in the blood reflected changes in the gut epithelial barrier and the Th17 compartment. To do this, we took advantage of animal models to measure DPP4 expression in distinct compartments of the gut. We compared *DPP4* mRNA expression in the pathogenic model of HIV, that is, rhesus macaques (MAC) infected by SIVmac, to the non‐pathogenic model, that is, AGM infected by SIVagm 63. AGM are known to preserve Th17 levels in the gut and to maintain an intact intestinal barrier despite high viraemia 23. The animals were infected with viral doses resulting in the same viraemia levels in both species (as shown for instance in the Figure S1 of reference 64). Gut biopsies were collected at day 65 post‐infection (p.i.). Four distinct gut compartments were analysed for each model corresponding to two compartments of the small intestine (ileum and jejunum) and two compartments from the large intestine (colon and rectum). Th17 cells are known to be most frequent in the small intestine 21, 31, 65. To measure *in situ* production, we quantified the mRNA expression. *DPP4* mRNA was generally higher in the CD4^+^ cell fraction as compared to CD4^−^ cells for both MAC and AGM (Figure S4A). In the AGM, DPP4 mRNA was higher in the small intestine than in the large intestine (Figure 3a). We then evaluated the correlation between plasma and intestinal *DPP4* in MAC and AGM. *DPP4* mRNA expression in the small intestine correlated with plasma DPP4 activity when values from the two distinct species were pooled (Figure 3b). Within each species, the correlations were not statistically significant, but we cannot exclude that the number of animals studied per species was too low to detect an association.

We next quantified *RORC* gene expression, a gene coding for the master transcription factor of Th17 cells, in the same samples (Figure 3c). *RORC* mRNA levels were higher in CD4^+^ cells of the small intestine than in those of the large intestine in AGM and higher in CD4+ than CD4‐ cells in the small intestine (Figures 3c , S4B). While *DPP4* and *RORC* gene expression profiles were similar to each other, *TBX21* mRNA showed a distinct profile from DPP4 and RORC (Figure 3e). *TBX21* mRNA was higher in CD4^+^ cells of the small than large intestine in MAC in contrast to *DPP4* and *RORC* that were not elevated. In line with this, *DPP4* and *RORC* mRNA expression in CD4^+^ cells positively correlated in the small intestine, while there was no correlation between *DPP4* and *TBX21* (Figure 3d,f). *RORC* mRNA in the small intestine also positively correlated with plasma sDPP4 activity (*R* = 0.67, *p* < 0.04). As for *DPP4* mRNA, the correlations were only statistically significant when the results of the two species were pooled. We focused the analysis on the small intestine, because this is the major site for Th17 cells, but Th17 cells can also be detected in the rectum 28, 31, 66. *DPP4* and *RORC* mRNA expression in CD4^+^ cells also positively correlated in the large intestine (*R* = 0.79, *p* < 0.05).

These results show that *DPP4* expressions were highest in the CD4^+^ cell fraction from the small intestine in AGM. The *DPP4* gene expression profiles were distinct from that of *TBX21* and rather resembled those of *RORC*. One possible explanation is that RORC^+^CD4^+^ cells, corresponding to Th17 cells, contribute to DPP4 production in the gut.

### 3.5 sDPP4 levels are increased in animals treated with IL‐21

**Figure 4:**
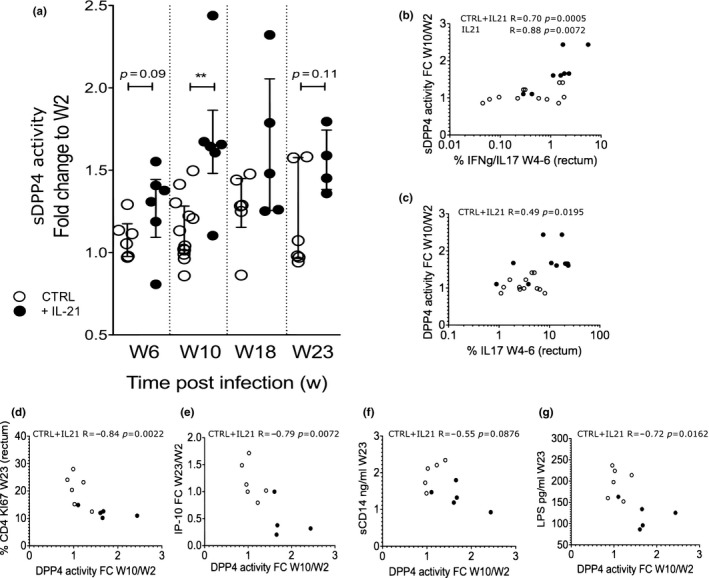
sDPP4 dynamics in blood after IL‐21 immunotherapy of SIV‐infected macaques. **(a)** sDPP4 activity in blood was measured in a longitudinal analysis before initiation of IL‐21 therapy (week 2 p.i.), at the end of IL‐21 treatment (week 6 p.i.) and after IL‐21 treatment (Weeks 10, 18 and 23 p.i.). The ratio of pre‐ and post‐treatment sDPP4 levels are shown. **(b and c)** IL‐17^+^ and IL‐17^+^ IFN‐•+ cells were measured in rectal biopsies on week 4 and week 6. **(b)** Correlation between the percentage of intestinal IL‐17^+^ IFN‐•+ cells and the plasma sDPP4 activity (fold change from week 2 to week 10) **(c)** Correlation between the percentage of intestinal IL‐17^+^ cells and plasma sDPP4 activity (fold change from week 2 to week 10). **(d‐f)** Correlation between sDPP4 activity in blood (fold change between week 2 and 10) and levels of **(d)** Ki67^+^ CD4^+^ T cells in gut **(e)** blood IP‐10 levels **(f)** blood sCD14 and **(g)** blood LPS after IL‐21 therapy cessation at week 23 p.i. For graph **a,** the Wilcoxon non‐parametric test was used; for graphs **b** to **g,** the Spearman non‐parametric correlation. The median and interquartile range are shown in graph **a**. *p < 0.05; **p < 0.01. Six rhesus macaques infected with SIVmac and treated with IL‐21 and six rhesus control macaques infected with SIVmac were analysed. CTRL: control monkeys; +IL‐21: IL‐21‐treated monkeys.

To further investigate links between sDPP4 blood levels and intestinal Th17 cells, we tested the hypothesis that animals showing increases in intestinal Th17 cells subsequent to an immunotherapy would also display increases in peripheral sDPP4. Pre‐clinical studies in the macaque model have shown promising results with IL‐21 treatment regarding the restoration of intestinal Th17 cells 28, 31, 66. We therefore quantified sDPP4 in plasma samples from SIV‐infected macaques that had been treated with IL‐21. These animals had received weekly rIL‐21 from week two p.i. for four weeks 31. Six macaques treated with IL‐21 and six control animals were analysed (Figure 4a). Peripheral sDPP4 levels indeed increased in the IL‐21‐treated animals. The animals showed a trend for sDPP4 increase at the end of the IL‐21 treatment (week 6 p.i) and a statistically significant increase at the following time point that was four weeks after IL‐21 administration (week 10 p.i, *p *< 0.01).

It has been previously described that the increase in Th17 cells in the gut of these animals were the strongest by the end of IL‐21 therapy, that is, at week 4‐6 p.i. 31. Th17 cells can be further categorized into Th17 (IL17^+^) and Th1Th17 (IFN‐γ^+^IL17^+^) cells. Since Th17 cells have been described to express the highest levels of DPP4 among CD4^+^ T cells 36, both cell populations (IL17^+^ and IFN‐γ^++^IL17^+^ CD4 T cells in the gut) were analysed. The percentages of IL17+ and IFN‐γ^+^IL17^+^ CD4^+^ T cells in the IL‐21‐treated animals were previously measured and published 31 and these values were used here to compare the correlations between IL‐17^+^ cells and sDPP4 levels at time points where the changes of IL‐17^+^ cells were reported to be statistically significant (week 4 and week 6). The frequencies of intestinal IL‐17^+^CD4^+^ and IL‐17^+^IFN‐g^+^CD4^+^ cells at these time points correlated with the subsequent increased levels of sDPP4 (Figure 4b,c).

Also, the previous study showed that immune activation and microbial translocation markers were markedly decreased at Week 23 (end of the study) in IL‐21‐treated animals 31. We used the previously 31 determined levels of these markers (intestinal Ki67^+^CD4^+^T cells, plasma sCD14, plasma LPS and plasma IP‐10) and analysed the correlation with sDPP4 levels. Interestingly, sDPP4 levels negatively correlated with immune activation markers (CD4^+^ T‐cell proliferation in the gut, plasma IP‐10 and plasma LPS, Figure 4d,e,g). There also was a trend for a negative correlation with sCD14 but this was not statistically significant (Figure 4f).

Together, these data indicate that IL‐21 therapy increased systemic sDPP4 activity. This sDPP4 increase could be indirect and a consequence of Th17 cell increases in the gut.

## 4 Discussion

DPP4 levels have been studied in many non‐communicable pathologies, and abnormal levels have been observed in metabolic disorders (i.e. type II diabetes and metabolic syndrome) 67, 68, auto‐immunity (i.e. multiple sclerosis, rheumatoid arthritis) 56, 69 and cancer (i.e. B‐cell leukaemia, hepatocellular carcinoma, bladder cancer) 61, 70, 71. In addition, DPP4 inhibitors are a widely used therapy for type II diabetes 72. In the setting of cancer, it has recently been proposed that DPP4 inhibitors could enhance trafficking of anti‐tumour immune cells into the lesions 73. DPP4 has been less studied in viral infections, with the exception of HCV 38, 74. A study on HIV infection provided evidence that immunomodulation by Ursodeoxycholic acid can increase the number of CD4^+^DPP4^+^ T cells in AIDS patients 62. Herein, we have studied for the first time sDPP4 levels in primary HIV infection, demonstrating that sDPP4 is severely decreased during PHI in two independent cohorts. sDPP4 levels were also lower in chronic HIV infection, as compared to healthy controls. This is in line with previous studies reporting decreased levels of sDPP4 in chronic HIV infection 43. Furthermore, we also report here DPP4 levels through a longitudinal analysis, starting prior to initial infection and revealing that the sDPP4 levels recovered only partially after PHI, and remained lower than in healthy donors.

Our study also reveals a strong association between plasma sDPP4 activity and disease progression. Thus, sDPP4 activity was most reduced during PHI in HIV‐infected patients who subsequently progressed rapidly. This was validated in two independent cohorts. Of note, the sDPP4 levels in PHI had a stronger predictive value for disease progression than viral load in PHI. However, as the difference in sDPP4 levels between rapid progressors and slow/normal progressors, although statistically significant, was small, measuring sDDP4 seems unlikely to be a useful measure in clinics of rapid disease progression in newly infected patients. We previously reported that plasma IP‐10 levels are increased in PHI, especially in rapid progressors 48. During PHI, IP‐10 levels were even more predictive of disease progression than RNA viraemia and CD4 T‐cell counts, while soluble CD163 levels were not. Here we analysed the same patients as in the previous studies 44, 48. Remarkably, sDPP4 levels in PHI were also more predictive than CD4 T‐cell counts, viraemia and total cell‐associated HIV DNA for rapid disease progression. However, the predictive values were lower than those for IP‐10. IP‐10 thus seems to be a better early marker for rapid disease progression than sDPP4.

Our animal studies allowed us to analyse DPP4 expression in the gut. *DPP4* mRNA expression was higher in the CD4+ than the CD4‐ fraction. Th17 cells have been described to express the highest levels of DPP4 among CD4^+^ T cells 36, but DPP4 is known to be also expressed by other intestinal cells, such as MAIT cells 75. We cannot exclude that the decrease in sDPP4 that we observed in the blood of the patients and the gut of the macaques was solely due to the loss of Th17 cells. However, *DPP4* mRNA expression was associated with *RORC* mRNA expression in CD4+ cells from the gut, supporting that the loss of DPP4 is at least partially related to dynamics of mucosal Th17 cells.

Studies with HIV‐1‐exposed but non‐infected female sex workers revealed that their resistance to HIV infection was associated with increased concentrations of blood sDPP4 and elevated sDPP4 expression (gene and protein level) in PBMCs 76, 77. The reasons for this are not well understood. In this context though it is interesting to note that SIV replication in the macaque is limited by the size of the pre‐existing Th17 cell compartment 78. Indeed, in that study it was shown that animals with a high level of Th17 cells in intestinal tissue before infection experienced lower viral load. DPP4 was not measured in that study and it is theoretically possible that the higher DPP4 levels in the exposed but non‐infected individuals reflected higher intestinal Th17 levels. Our studies in IL‐21 treated, SIV‐infected animals further support the link between sDPP4 in blood and intestinal Th17 levels. Here, DPP4 levels were increased after IL‐21 therapy in the animal model and correlated with increases in intestinal Th17 cells.

It was striking to observe that in patients under cART, the sDPP4 activity levels did not increase. This contrasts with a study reporting that cART leads to a gain of plasma sDPP4 activity 43. This study however included only two patients, none of whom received a protease inhibitor. Future studies are needed to evaluate whether anti‐HIV protease inhibitors influence sDPP4 activity, as compared with other anti‐retroviral regimen such as integrase inhibitors. In case anti‐HIV protease inhibitors would impact DPP4 activity, we cannot exclude that residual protease inhibitors within the plasma samples were affecting the enzyme activity assay. This has implications if sDDP4 is to be used as a surrogate marker to determine if new therapies are improving the recovery of the gastrointestinal tract in individuals receiving PI‐cART. In the patients here, cART was initiated only in the chronic phase and relatively recently (median 34 months) 46, 47. It is thus possible that sDPP4 levels did not increase because cART in these chronically HIV‐infected, recently treated patients did not restore gut Th17 cells.

Soluble DPP4 is already an established biomarker in other diseases such as type II diabetes 67, 68. We propose that sDPP4 could be used as a systemic surrogate marker for evaluation of the capacity of potential therapies to restore gut immune function, in particular Th17 levels in the gut during HIV infection. A curative strategy of HIV will likely need to both reduce the amount of virus that persists on anti‐retroviral therapy and improve anti‐HIV immune surveillance 15, 79. Altogether, our study reveals the potential benefit of using sDPP4 as a biomarker in the field of HIV cure/remission and paves the way for future studies aimed at evaluating its potential use as a biomarker for restoration of gut immunity.

## Authors contributions

MJP and AC performed the ELISA experiments. MJP performed the qPCR assays. MJP, BJ, NH, SJP and TGT performed the intestinal tissue preparations. CL, MG, OL, CG and NK provided human samples. RLG, LM and MP provided simian samples. FB, TB, MG, NK and LM performed data curation regarding samples from the human cohorts. AC and DD developed tools. MJP, DD, MA and MMT developed the concept of the study and its experimental design. All authors participated in the analyses and interpretation of the data. MJP, YM and NN performed the statistical analyses and LM supervised them. MJP, NN and YM prepared the figures. BJ wrote the animal protocols. MMT wrote the human protocols, supervised the research and wrote the manuscript, which was reviewed by the co‐authors.

## Supporting information


**Figure S1.** Sequences of the PCR amplicons for three species (human, AGM, MAC).
**Figure S2.** Comparison of enzymatic activity of sDPP4 in plasma compared to the absolute concentration of sDDP4 qualified in the same samples.
**Figure S3.** Soluble DPP4 activity and concentration in blood during primary HIV‐1 infection in patients with distinct disease progression profiles.
**Figure S4.** Levels of *DPP4* and *RORC* mRNA in CD4^+^ and CD4^−^ cells of the gut.

**Table S1.** Characteristics of the patients from the four distinct cohorts.
**Table S2.** Comparison of the levels of sDDP4 in HIV controllers (HIC) compared to those in healthy donors (HD) and other HIV‐infected individuals.
