# Supplementary material for: Systemic DPP4 activity is reduced during primary HIV‐1 infection and is associated with intestinal RORC + CD4+ cell levels: a surrogate marker candidate of HIV‐induced intestinal damage
Source: J Int AIDS Soc. 2018 Jul 10;21(7):e25144. doi: 10.1002/jia2.25144 (PMC6038000; doi:10.1002/jia2.25144)
Supplement: Supplementary file 1 — Figure S1. Sequences of the PCR amplicons for three species (human, AGM, MAC). Figure S2. Comparison of enzymatic activity of sDPP4 in plasma compared to the absolute concentration of sDDP4 qualified in the same samples. Figure S3. Soluble DPP4 activity and concentration in blood during primary HIV‐1 infection in patients with distinct disease progression profiles. Figure S4. Levels of DPP4 and RORC mRNA in CD4+ and CD4− cells of the gut. [file JIA2-21-e25144-s001.pdf]

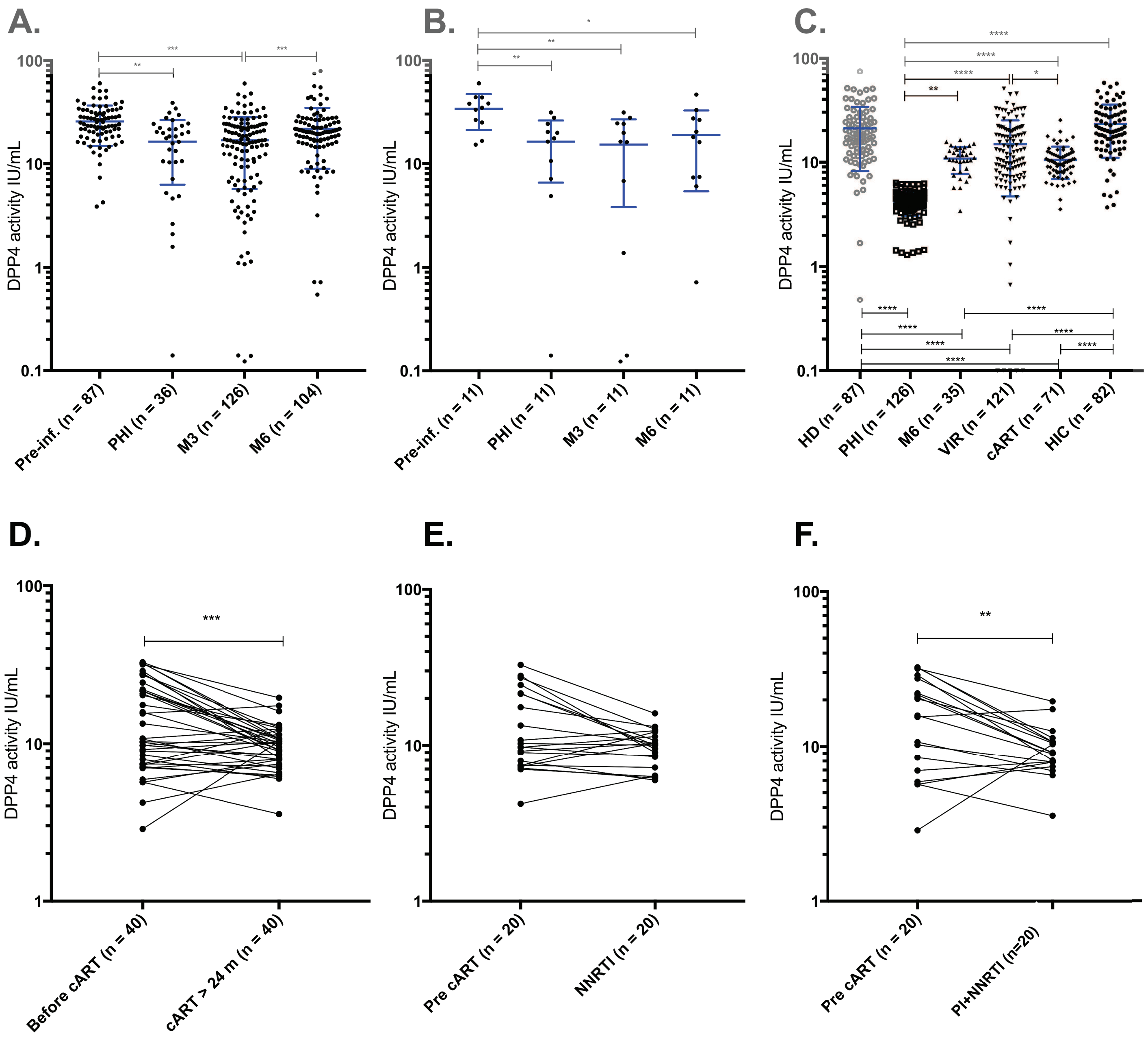
A.
B.
C.
D.
E.
F.
DPP4 activity IU/mL
Pre-inf. (n = 87)
PHI (n = 36)
M3 (n = 126)
M6 (n = 104)
Pre-inf. (n = 11)
PHI (n = 11)
M3 (n = 11)
M6 (n = 11)
HD (n = 87)
PHI (n = 126)
M6 (n = 35)
VIR (n = 121)
cART (n = 71)
HIC (n = 82)
Before cART (n = 40)
cART > 24 m (n = 40)
Pre cART (n = 20)
NNRTI (n = 20)
PI+NNRTI (n=20)

Figure 1

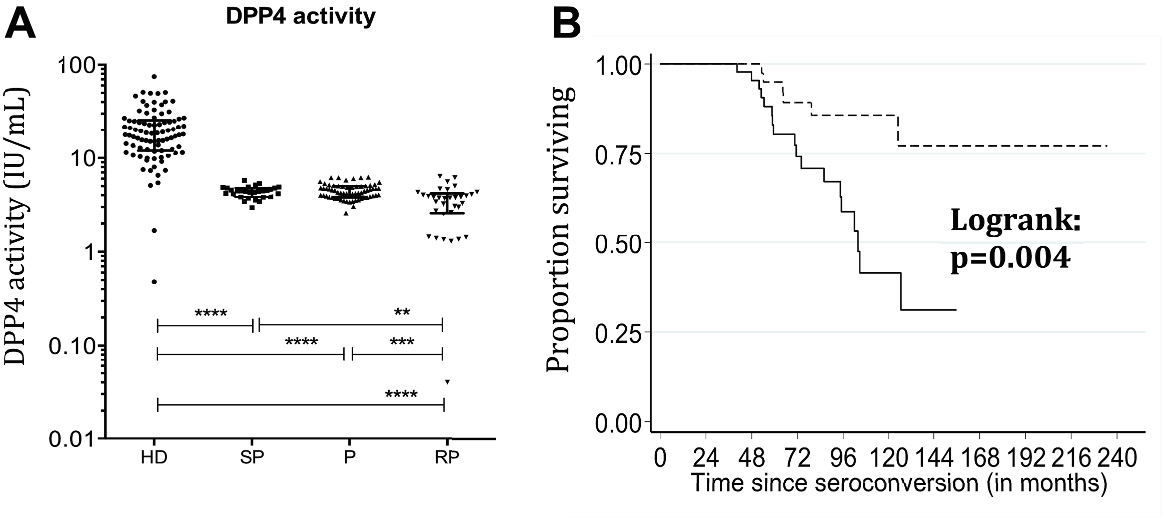

A
DPP4 activity
DPP4 activity (IU/mL)
100
10
1
0.10
0.01
****
**
****
***
****
HD
SP
P
RP
B
Proportion surviving
1.00
0.75
0.50
0.25
0.00
Logrank:
p=0.004
0 24 48 72 96 120 144 168 192 216 240
Time since seroconversion (in months)

Figure 2

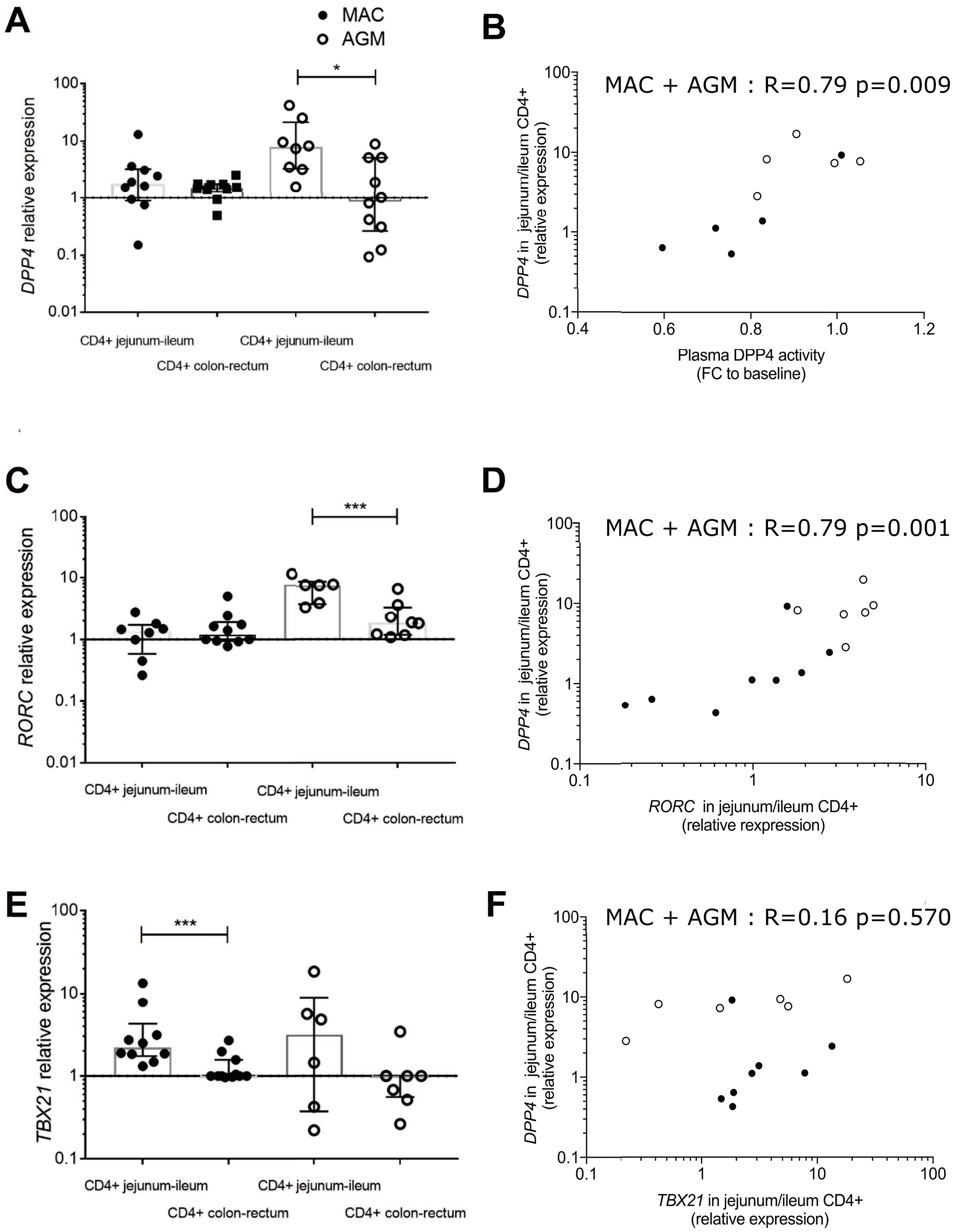
A
MAC
AGM
*
DPP4 relative expression
100
10
1
0.1
0.01
CD4+ jejunum-ileum
CD4+ colon-rectum
CD4+ jejunum-ileum
CD4+ colon-rectum
B
MAC + AGM : R=0.79 p=0.009
DPP4 in jejunum/ileum CD4+ (relative expression)
Plasma DPP4 activity (FC to baseline)
0.4
0.6
0.8
1.0
1.2
C
***
RORC relative expression
D
MAC + AGM : R=0.79 p=0.001
RORC in jejunum/ileum CD4+ (relative rexpression)
E
***
TBX21 relative expression
F
MAC + AGM : R=0.16 p=0.570
TBX21 in jejunum/ileum CD4+ (relative expression)

Figure 3

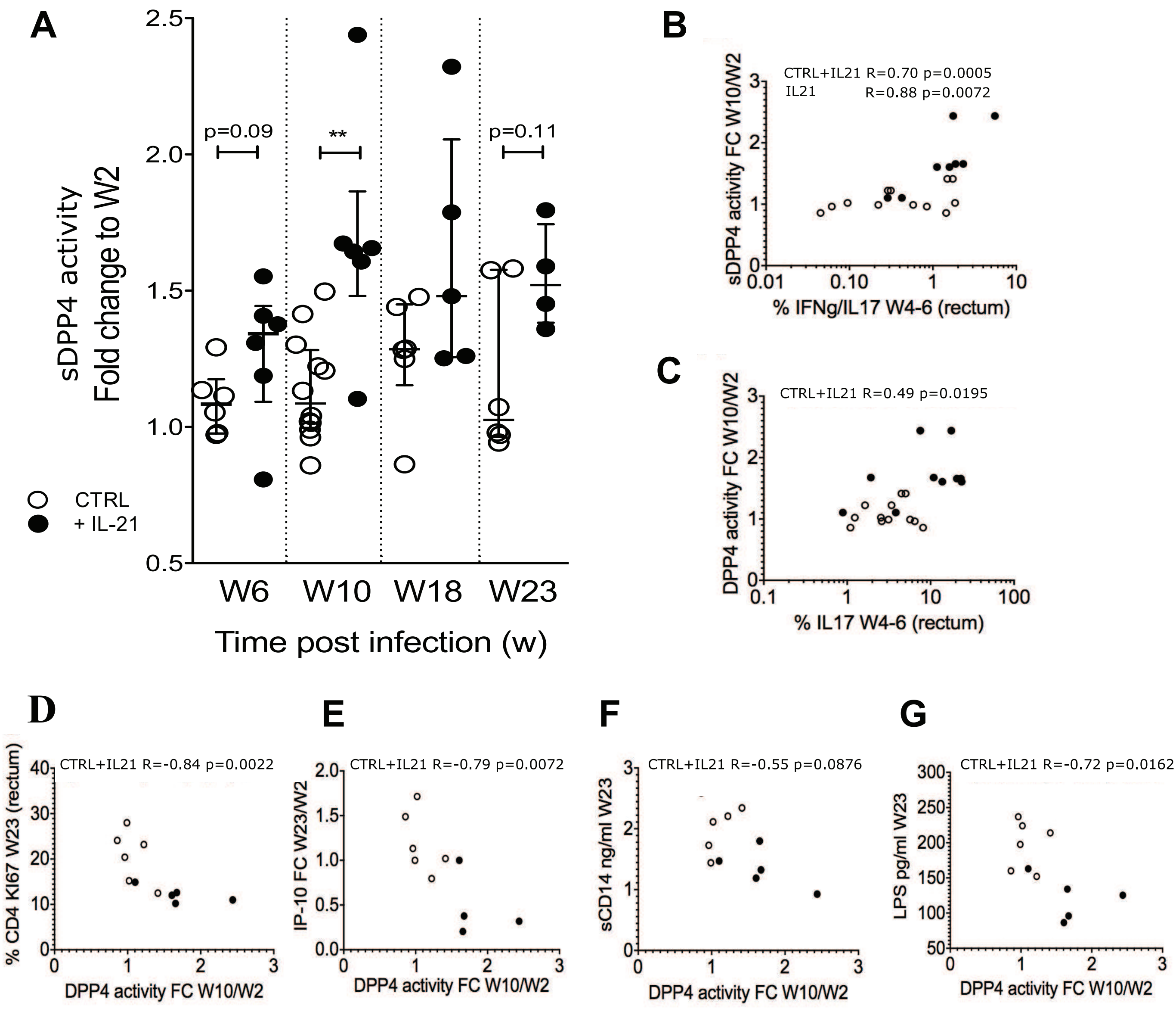
A
sDPP4 activity
Fold change to W2
p=0.09
**
p=0.11
CTRL
+ IL-21
W6
W10
W18
W23
Time post infection (w)
B
CTRL+IL21 R=0.70 p=0.0005
IL21 R=0.88 p=0.0072
sDPP4 activity FC W10/W2
% IFNg/IL17 W4-6 (rectum)
C
CTRL+IL21 R=0.49 p=0.0195
DPP4 activity FC W10/W2
% IL17 W4-6 (rectum)
D
CTRL+IL21 R=-0.84 p=0.0022
% CD4 KI67 W23 (rectum)
DPP4 activity FC W10/W2
E
CTRL+IL21 R=-0.79 p=0.0072
IP-10 FC W23/W2
DPP4 activity FC W10/W2
F
CTRL+IL21 R=-0.55 p=0.0876
sCD14 ng/ml W23
DPP4 activity FC W10/W2
G
CTRL+IL21 R=-0.72 p=0.0162
LPS pg/ml W23
DPP4 activity FC W10/W2

Figure 4

A) DPP4

>Hum DPP4
GAGGAAGTGGTTACCAAGGAGATAAGATCATGCATGCAATCAACAGAAGACTGGGAACATTTGAAGTTGAAGATC
AAATTGAAGCAGCCAGACAATTTTCAAAAATGGGATTTGTGGACAACAAACGAATTGCAATTTGGGGCTGGTCAT
ATGGAGGGTACGTAACCTCAATGGTCCTGGG

>AGM DPP4
GAGGAAGTGGTTACCAAGGAGATAAGATCATGCATGCAATCAACAGAAGACTGGGAACATTTGAAGTTGAAGATC
AAATTGAAGCAGCCAGACAATTTTCAAAAATGGGATTTGTGGACAACAAACGAATTGCAATTTGGGGCTGGTCAT
ATGGAGGGTACGTAACCTCAATGGTCCTGGG

>MAC DPP4
GAGGAAGTGGTTACCAAGGAGATAAGATCATGCATGCAATCAACAGAAGACTGGGAACATTTGAAGTTGAAGATC
AAATTGAAGCAGCCAGACAATTTTCAAAAATGGGATTTGTGGACAACAAACGAATTGCAATTTGGGGCTGGTCAT
ATGGAGGGTACGTAACCTCAATGGTCCTGGG

B) TBX21

>Hum TBX21
TGTTGTGGTCCAAGTTTAATCAGCACCAGACAGAGATGATCATCACCAAGCAGGGACGGCGGATGTTCCCATTCC
TGTCATTTACTGTGGCCGGGCTGGAGCCCACCAGCCACTACAGGATGTTT

>AGM TBX21
TGTTGTGGTCCAAGTTTAATCAGCACCAGACAGAGATGATCATCACCAAGCAGGGACGGCGGATGTTCCCATTCC
TGTCATTTACGGTGGCTGGGCTGGAGCCCACTAGCCACTACAGGATGTTT

>MAC TBX21
TGTTGTGGTCCAAGTTTAATCAGCACCAGACAGAGATGATCATCACCAAGCAGGGACGGCGGATGTTCCCATTCC
TGTCATTTACGGTGGCTGGGCTGGAGCCCACTAGCCACTACAGGATGTTT

C) RORC

>Hum RORC
AGCTGGCCTTTCATCATCATCTCTGCAAGACTCATCGCCAAAGCATCCTGGCAAAGCTGCCACCCAAGGGGAAGC
TTCGGAGCCTGTGTAGCCAGCATGTGGAAAGGCTGCAGATCTTCCAGCAC

>AGM RORC
AGCTGGCATTTCATCATCATCTCTGCAAAACTCATCGCCAAAGCATCCTGGCAAAGCTGCCACCCAAGGGGAAGC
TTCGGAGCCTGTGTAGCCAGCACATGGAAAGGCTGCAGATCTTCCAGCAC

>MAC RORC
AGCTGGCCTTTCATCATCATCTCTGCAAAACTCATCGCCAAAGCATCCTGGCAAAGCTGCCACCCAAGGGGAAGC
TTCGGAGCCTGTGTAGCCAGCACGTGGAAAGGCTGCAGATCTTCCAGCAC

D) 18S

>Hum 18S
CAGGGTTCGATTCCGGAGAGGGAGCCTGAGAAACGGCTACCACATCCAAGGAAGGCAGCAGGCGCGCAAATTACC
CACTCCCGACCCGGGGAGGTAGTGACGAAAAATAACAATACAGGACTCTTTCGAGGCCCTGTAATTGGAATGAGT
CCACTTTAAATCCTTTAACGAGGATCCATTGGAGGGCAAGTCTGGTGCCAGCAGCCGCGGTAATTCCAGCTCCAA
TAGCGTATATTAAAGTTGCTGCAGTTAAAAAGCTCGTAGTTGGATCTTGGGAGCGGGCGGGCGGTCCGCCGCGAG
GCGAGCCACCGCCCGTCCCCGCCCCTTGCCTCTCGGCGCCCCCTCGATGCTCTTAGCTGAGTGTCCCGCGGGGCC

>MAC
CAGGGTTCGATTCCGGAGAGGGAGCCTGAGAAACGGCTACCACATCCAAGGAAGGCAGCAGGCGCGCAAATTACC
CACTCCCGACCCGGGGAGGTAGTGACGAAAAATAACAATACAGGACTCTTTCGAGGCCCTGTAATTGGAATGAGT
CCACTTTAAATCCTTTAACGAGGATCCATTGGAGGGCAAGTCTGGTGCCAGCAGCCGCGGTAATTCCAGCTCCAA
TAGCGTATATTAAAGTTGCTGCAGTTAAAAAGCTCGTAGTTGGATCTTGGGAGCGGGCGGGCGGGCGGTCCGCCG
CGAGGCGAGCCACCGCCCGTCCCCGCCCCCTTGCCTCTCGGCGCCCCCTCGATGCTCTTAGCTGAGTGTCCCGCG
GGGCC

Supplementary Figure 1

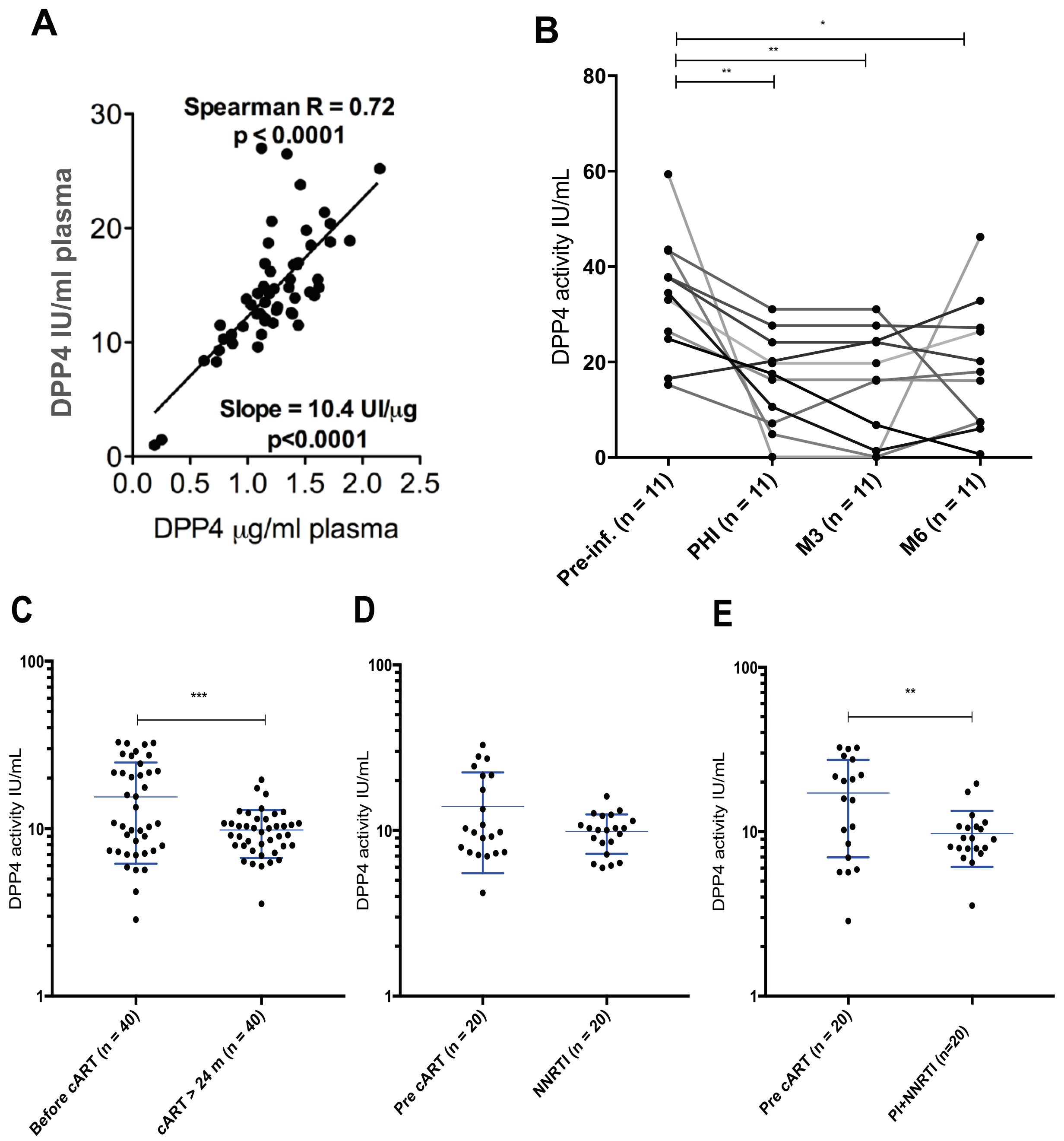

Supplementary Figure 2

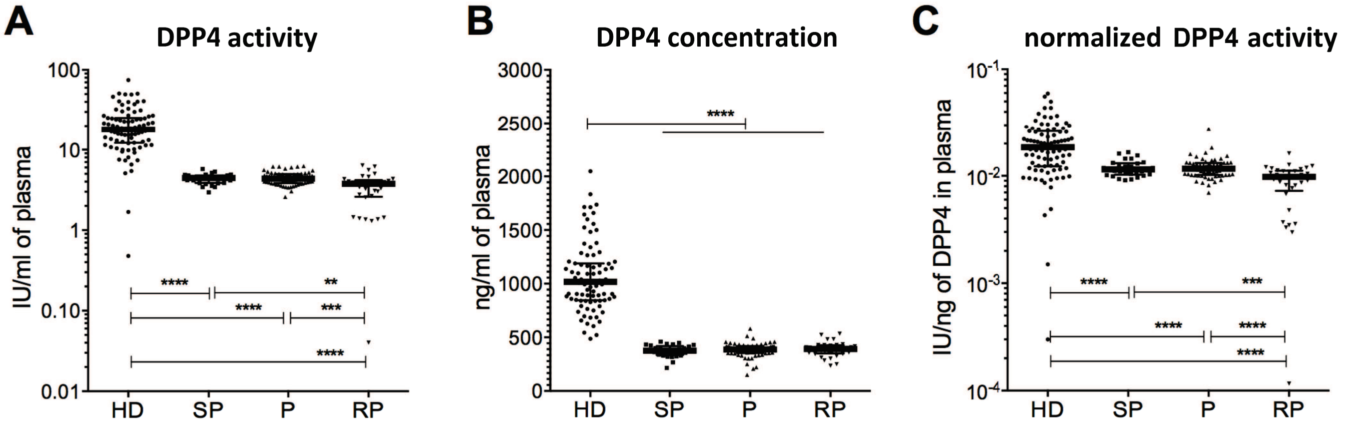

Supplementary Figure 3

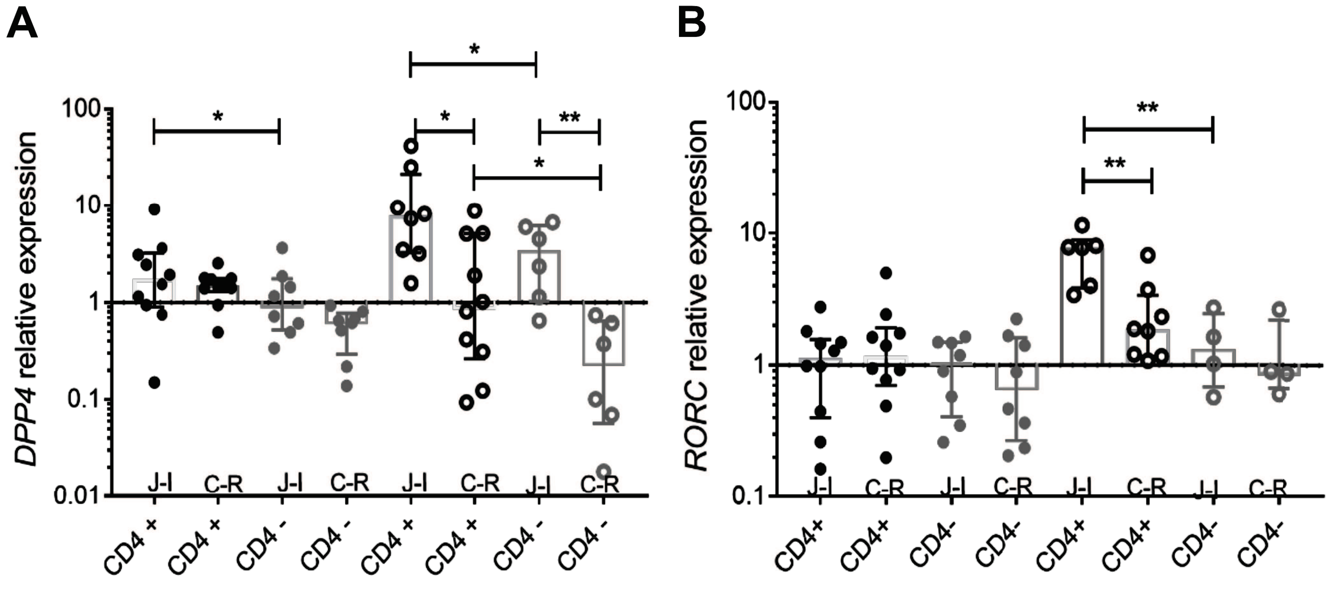

Supplementary Figure 4
