# Supplementary material for: Systemic DPP4 activity is reduced during primary HIV‐1 infection and is associated with intestinal RORC + CD4+ cell levels: a surrogate marker candidate of HIV‐induced intestinal damage
Source: J Int AIDS Soc. 2018 Jul 10;21(7):e25144. doi: 10.1002/jia2.25144 (PMC6038000; doi:10.1002/jia2.25144)
Supplement: Supplementary file 2 — Table S1. Characteristics of the patients from the four distinct cohorts. Table S2. Comparison of the levels of sDDP4 in HIV controllers (HIC) compared to those in healthy donors (HD) and other HIV‐infected individuals. [file JIA2-21-e25144-s002.docx]

**Systemic DPP4 activity is reduced during primary HIV-1 infection and is associated with intestinal RORC^+^CD4^+^ cell levels: a surrogate marker candidate of HIV-induced intestinal damage**

Mickaël J. Ploquin^1^, Armanda Casrouge^2,3^, Yoann Madec^4^, Nicolas Noël^1,5,6,7^, Beatrice Jacquelin^1^, Nicolas Huot^1^, Darragh Duffy^2,3^, Simon P. Jochems^1$^, Luca Micci^8^, Camille Lécuroux^6^, Faroudy Boufassa^9^, Thijs Booiman^10^, Thalia Garcia-Tellez^1^, Mathilde Ghislain^9^, Roger Le Grand^6^, Olivier Lambotte^5,6,7^, Neeltje Kootstra^10^, Laurence Meyer^7,9^, Cecile Goujard^5,7,9^, Mirko Paiardini^8^, Matthew L. Albert^2,*^ & Michaela Müller-Trutwin^1,§^.

^1^Institut Pasteur, Unité HIV, Inflammation et Persistance, Paris, France, ^2^Institut Pasteur, Unité Immunobiologie des cellules dendritiques, Paris, France, ^3^INSERM U1223, Paris, France, ^4^Institut Pasteur, URE Epidémiologie des Maladies Emergentes, Paris, France, ^5^Assistance Publique – Hôpitaux de Paris, Service de Médecine Interne et Immunologie Clinique, Groupe Hospitalier Universitaire Paris Sud, Hôpital Bicêtre, Le Kremlin-Bicêtre, France, ^6^CEA, Université Paris Sud, Inserm U1184, Immunology of viral infections and auto-immune diseases (IMVA), IBFJ, IDMIT Department, Fontenay-aux-Roses and Kremlin-Bicêtre, France ^7^Université Paris Sud, Le Kremlin Bicêtre, France, ^8^Emory University School of Medicine and Yerkes National Primate Research Center, Atlanta, Georgia, USA, ^9^INSERM CESP U1018, Université Paris Sud, Le Kremlin-Bicêtre, France, ^10^Academisch Medisch Centrum, Laboratory of Viral Immune Pathogenesis, Amsterdam The Netherlands

$ Current address: Liverpool School of Tropical Medicine, Liverpool, UK

*** Current address: Department of Cancer Immunology, Genentech Inc. California, USA

^§^ Corresponding author

Short title: DPP4 dynamics in blood and gut during HIV/SIV infections

Keywords: HIV, SIV, inflammation, intestine, Dipeptidylpeptidase, biomarker, Th17

COUNTS: Abstract: 349 words, Manuscript: 5864 words, 80 References, 1 Table, 4 Figures, 2 supplementary Tables, 4 Supplementary Figures

**Abstract**

***Introduction:*** Combined anti-retroviral therapy (cART) transformed HIV-1 from a deadly disease into a chronic infection, but does not cure HIV infection. It also does not fully restore HIV-induced gut damage unless administered extremely early after infection. Additional biomarkers are needed to evaluate the capacity of therapies aimed at HIV remission/cure to restore HIV-induced intestinal immune damage and limit chronic inflammation. Herein, we aimed to identify a systemic surrogate marker whose levels would reflect gut immune damage such as intestinal Th17 cell loss starting from primary HIV-1 infection.

***Methods:*** Biomarker discovery approaches were performed in four independent cohorts, covering HIV-1 primary and chronic infection in 496 naïve or cART-treated patients (Amsterdam cohort (ACS), ANRS PRIMO, COPANA and CODEX cohorts). The concentration and activity of soluble Dipeptidylpeptidase 4 (sDPP4) were quantified in the blood from these patients, including pre- and post-infection samples in the ACS cohort. For quantification of DPP4 in the gut, we utilized two non-human primate models, representing pathogenic (macaque) and non-pathogenic (African green monkey) SIV infection. Four gut compartments were analyzed in each animal model (ileum, jejunum, colon and rectum) for quantification of *DPP4*, *RORC*, and *TBX21* gene expression in sorted CD4^+^ cells. To analyze if sDPP4 levels increase when Th17 cells were restored, we quantified sDPP4 in plasma from SIV-infected macaques treated with IL-21.

**Results:** We showed that sDPP4 levels were strongly decreased in primary HIV-1 infection. Strikingly, sDPP4 levels in primary HIV-1 infection predicted time to AIDS. They were not increased by cART in chronic HIV-1 infection (median 36 months on cART). In the gut of SIV-infected non-human primates, *DPP4* mRNA was higher in CD4^+^ than CD4^-^ leukocytes. DPP4 specifically correlated with RORC expression, a Th17 marker, in CD4^+^ cells from the intestine. We further demonstrated that sDPP4 activity levels were increased in animals treated with IL-21 and that this increase was associated with restoration of the Th17 compartment and reduced inflammation. Furthermore, *DPP4* mRNA levels in small intestine CD4^+^ cells positively correlated with circulating DPP4 activity.

***Conclusion:*** These data provide evidence that blood sDPP4 levels could be useful as a correlate for HIV-induced intestinal damage.

**Introduction**

Combined anti-retroviral treatment (cART) drastically improves life expectancy and quality of life of HIV-infected individuals [1,2] but does not result in viral cure. Despite sustained undetectable viremia during efficient cART, the virus persists in so-called viral reservoirs [3–5]. In addition, a residual chronic inflammation persists in treated patients [6–9]. Indeed, while cART lowers chronic inflammation concomitantly with a reduction in viral load, in most patients the inflammation levels are not normalized and remain higher than in healthy persons [6,8]. Even in spontaneous HIV controllers (HIC), the inflammation level remains most often higher as compared to healthy donors and those HIC with higher inflammatory scores have a higher risk for CD4+ T cell loss and disease progression [10,11]. Of note, the residual and persistent inflammation under effective cART is associated with a higher risk of comorbidities and non-AIDS illnesses, such as cancer and cardiovascular diseases [12,13].

A therapy leading to complete viral eradication [3,14], or with higher likelihood to long-term undetectable viremia in the absence of cART [15], is the aspirational goal. This ideal scenario would allow treatment cessation without an increased risk of transmission to partners, while also reducing inflammation and fully restoring immune function and tissue integrity, reducing the risk of increased co-morbidities and non-AIDS mortality.

In the absence of cART, HIV-1 infection induces a rapid depletion of CD4^+^ T cells in the gut, in particular Th17 cells, and epithelial barrier disruption ensues along with associated microbial translocation contributing to chronic inflammation [16–18]. It has been shown that cART initiation at the earliest stages of HIV-1 infection (Fiebig stages I and II), prevents Th17 cell depletion and fully reverses the initially observed mucosal and systemic immune-activation [19,20]. By contrast, cART initiation at Fiebig stage III restored Th17 cell numbers, but not their polyfunctionality, and patients maintained elevated mucosal and systemic CD8^+^ T-cell activation post initiation of cART [19]. In the majority of the cases, cART is initiated during chronic HIV-1-infection. It generally results in good immune reconstitution in peripheral blood, however reconstitution in the gut occurs at a much slower pace, where both immunological and structural abnormalities persist even in long-term treated patients [21]. Of particular interest are therapeutic approaches aimed at reversing intestinal damage [22–27]. It is unclear so far if one of these agents could be useful, although a few pilot studies provided some promising results. Treatment with Mesalamine, which is used in clinics to decrease mucosal inflammation in ulcerative colitis, did not change the persistent T cell activation in rectum nor plasma sCD14, IL-6, D-dimer levels during HIV-infection [22]. Rifaximin, a nonabsorbable antibiotic that decreases LPS in cirrhotics, had a marginal impact on microbial translocation in patients on cART [25]. Treatment with the drug Sevelamer, which binds microbial lipopolysaccharide in the gut, seems to confer cardiovascular benefits but its capacity to block microbial translocation is unclear [24]. In several studies, probiotic therapy or therapy with probiotics supplemented with IL-21 showed promising effects, such as enhanced CD4+ T cell restoration in the gut and/or decrease of inflammation and microbial translocation [28–30]. IL-21 alone resulted in transient increases of rectal Th17 cells and decrease in systemic inflammation [31]. To guide drug development in this area, systemic biomarkers evaluating the restoration of gut integrity and immune function are urgently required. Several systemic markers of gut epithelial barrier disruption are currently used in the field of HIV [18,32–34]. These markers include I-FABP, sCD14, LBP, LPS and bacterial RNA, the latter two being a direct measurement of microbial translocation [35]. While the use of these markers has helped the field to make incredible progress in the understanding of HIV physiopathology, few are entirely specific for microbial translocation or epithelial disruption in the gut (particularly sCD14, related to monocyte activation) and some are difficult to measure. Also, none of these biomarkers serve as a surrogate for intestinal Th17 cell levels in the gut.

Th17 cells have been described to express the highest levels of Dipeptidylpeptidase 4 (DPP4) among CD4^+^ T cells [36]*.* DPP4 is a membrane-associated enzyme (also known as CD26), which can exist in soluble form in blood. It cleaves hormones and chemokines such as Glucagon-like-peptide, SDF-1, all three CXCR3-ligands (IP-10, MIG, I-TAC), RANTES, CCL11 and CCL20 [37]. It was previously reported to be increased in hepatitis C (HCV) infection, acting to inhibit trafficking of CXCR3^+^ leukocytes to the sites of viral replication and possibly accounting for viral immune escape [38]. In contrast to HCV, a loss of circulating DPP4^+^CD4^+^ T cells and reduced plasma soluble DPP4 (sDPP4) activity has been observed during chronic HIV Infection [39–43]. Here, we examined whether sDPP4 levels in the blood can be used as a systemic marker of intestinal Th17 levels and gut immune damage during HIV infection.

**Methods**

### Ethics statement

Patient enrollment respected all European guidelines and those established by the World Medical Association in its declaration of Helsinki. All patients were adult subjects and gave their written informed consent. The scientific board of the Amsterdam cohort studies (ACS) approved this study. Concerning the French ANRS cohorts, the Paris-Cochin Ethics Committee approved the study protocols for the patients from PRIMO C06 and COPANA C09 cohorts, and the Comité de Protection des Personnes Ile-de-France VII, Paris, France (approval reference: 05–22) approved the CODEX CO21 study protocol. The Institut Pasteur “Comité de recherché Clinique” (CoRC #2013-05) equally approved this study.

Animals were housed in the facilities of the CEA (“Commissariat à l'Energie Atomique”, Fontenay-aux-Roses, France) IDMIT facilities (Infrastructure for Infectious Disease Models and Innovative Therapies), Fontenay-aux-Roses, France (permit number A 92-032-02) or the Institut Pasteur, Paris, France (permit number A 78-100-3). All experimental procedures were conducted in strict accordance with the European guideline 2010/63/UE on the protection of animals used for scientific purposes (French decree 2013-118) and with the recommendations of the Weatherall report. The monitoring of the animals was under the supervision of the veterinarians in charge of the animal facilities. All efforts were made to minimize suffering, including efforts to improve housing conditions and to provide enrichment opportunities in the IDMIT center (e.g., 12∶12 light dark schedule, provision of monkey biscuits supplemented with fresh fruit and constant water access, objects to manipulate, interaction with caregivers and research staff). All procedures were performed under anesthesia using 10 mg of ketamine per kg body weight. Euthanasia was performed prior to the development of any symptoms of disease. Euthanasia was done by IV injection of a lethal dose of pentobarbital. The CEA is in compliance with Standards for Human Care and Use of Laboratory of the Office for Laboratory Animal Welfare (OLAW, USA) under OLAW Assurance number #A5826-01. Animal experimental protocols were approved by the Ethical Committee of Animal Experimentation (CETEA-DSV, IDF, France; Notification number: 10-051b).

The analyses of the human and animal study samples took place between 2013 and 2016.

**Patient cohorts and samples**

A large serum library derived from cART-naïve patients, including pre-infection samples from patients enrolled in the Amsterdam Cohort Studies (ACS) of HIV infection and AIDS was used (Supplementary Table 1, [44]). None of the 136 subjects studied here had started antiretroviral therapy when samples were collected. These patients had an estimated date of seroconversion (SC) defined as the midpoint between the date of the last visit with a negative HIV test and the first visit with a positive HIV test (complete or incomplete western blot) [45]. Samples collected before infection were obtained between 24 and 3 months before the estimated date of SC. Patients co-infected with other bloodborn pathogens (HIV-2, HBV, HCV) were excluded.

The viremic cART-naive subjects (VIR, n=121) were part of the French ANRS C09 COPANA cohort (Supplementary Table 1) [44,46]. A subgroup from the COPANA Cohort of 40 patients received cART (>24 months of follow-up and >12 months with VL < 50 copies/mL) [47]. In addition, 31 randomly selected cART treated patients were analyzed. The median time on cART was 36 months (IQR, 27-66 months).

The HIC (n=82) were enrolled in the French ANRS C021 CODEX cohort (Supplementary Table 1, [44]). To be enrolled, patients had to be diagnosed as HIV-infected for more than 5 years and to remain cART-naive with viremia below 400 copies/ml in at least five consecutive assays, regardless of their CD4 cell count [10].

The 126 subjects enrolled in the French ANRS C06 PRIMO cohort were previously described [48]. Primary HIV infection (PHI) (M0) was defined by an incomplete WB, with detectable HIV RNA load and/or P24 protein. Most patients in PHI were in Fiebig stage III/IV. Patients were categorized as rapid progressors (RP), slow progressors (SP) or normal progressors (P) as previously described [48]. Briefly, RP were defined as those losing their peripheral CD4+ T cells to below 350 cells/mm^3^ after PHI in less than 12 months; P showed more than 350 CD4^+^ T cells/mm^3^ after 12 months but less than 500 cells/mm^3^ at least at one time point before or at 42 months post-PHI, in the absence of treatment; and SP were displaying more than 500 CD4+ T cells/mm^3^ after 42 months post-PHI in the absence of treatment. The cell-associated viral DNA load has been extensively studied in these patients [49–52].

None of the participants in the ANRS cohorts who we studied received immunosuppressive drugs, IFN therapy or chemotherapy, and none had cancer, autoimmune diseases or HIV-unrelated chronic inflammatory metabolic disorders at enrollment.

Frozen plasma from blood collected on EDTA was obtained from the ANRS cohorts.

EDTA plasma from HIV/HBV/HCV-seronegative individuals (Healthy Donors (HD), n=87) were obtained from *Etablissement Français du Sang* (EFS, Paris, France) for research purposes.

As mentioned above, in the ACS cohort, the patients with HCV infection were excluded. In the ANRS PRIMO and COPANA cohorts, only 2 individuals per cohort were HCV infected. In the 82 patients from the Codex cohort, 19 were HCV-infected (23%).

**Non-human primates**

Five Chinese rhesus macaques (*Macaca mulatta*) were I.V. infected with 50 AID_50_ (animal infectious dose 50) of an uncloned SIVmac251 isolate (provided by A. M. Aubertin, Université Louis Pasteur, Strasbourg, France). Twelve Indian rhesus macaques were I.V. infected with 300 TCID_50_ (tissue culture infectious dose 50). SIVmac_239_ as previously reported [31]. Five African green monkeys (*Chlorocebus sabaeus,* AGM) were I.V. infected with 250 TCID_50_ of purified SIVagm.sab92018 wildtype isolate [53–55]. Plasma was obtained from blood collected on EDTA. Intestinal samples from the AGM and the Chinese rhesus macaques were collected at sacrifice and treated as described below in the following paragraph. The longitudinal collection and treatment of the rectal samples from the Indian rhesus macaques have been previously reported [53–55].

**Isolation of CD4^+^ and CD4^-^ cells from simian gut tissue**

At necropsy (day 65 post-infection), a fragment (5/7cm in length) was collected from sections of the intestine (jejunum, ileum, colon and rectum) of all 5 Chinese rhesus macaques and 5 AGM, except for the ileum fragment that was collected in 3/5 macaques and 3/5 AGMs. The fragments were enzymatically dissociated in RPMI culture medium (*Life Technologies*) containing collagenase (Collagenase II-S, *Sigma-Aldrich*) and DNAse (*Sigma-Aldrich*) for 1 h with agitation (80 rpm) at 37°C. Total leukocytes were separated from epithelial/endothelial cells through a Percoll gradient. CD4 positive leukocytes were purified with magnetic beads (*Miltenyi* columns and CD4 cell purification kit) and the CD4 negative fraction collected.

**DPP4 assays**

The concentrations of sDPP4 were measured with the commercial sandwich ELISA (human DPP4/DPP4 Duo Set ELISA, R&D Systems) following the supplier's instructions, as described [38]. Briefly, Maxisorp plates (Nunc) were coated with 2 μg/mL of the commercial capture antibody in PBS overnight at room temperature. The wells were saturated with 300 μL of 1% in PBS for 1 h. A seven-point standard curve was performed with the highest concentration at 2000 pg/mL. Plasma samples were used at 1/800 dilution. Plates were analyzed in a Labsystems Multiskan MS (Thermo) device.

The sDPP4 enzymatic activity was measured using a luciferase-based assay (DPP4-Glo™ Protease Assay; Promega). This assay utilizes a luminogenic DPP substrate, Gly-Pro-aminoluciferin. After cleavage of the proximal two amino acids from the substrate, the aminoluciferin is free to engage luciferase. Relative luminescent units (RLU) are proportional to the DPP activity. This assay was performed on plasma samples serially diluted between 0.025%, 0.25% and 2.5% in 10 mm Tris–HCL pH 8 with 0.1% Prionex stabilizer (Calbiochem). The enzymatic activity was measured following the manufacturer's instructions, as previously described [38, 59, 62]. Briefly, in a white plate (Greiner bio-one), 50 μL of kit reagent containing the luciferase substrate was added to 50 μL of sample. Maximal signal is reached within 30 minutes and is stable for 3 hours. Recombinant human DPP4 (Sigma D4943) was used as a reference. A seven point standard curve, using 2-fold serial dilutions and duplicates of each dilution was applied. DPP activity was expressed in units/ml based on supplier information after subtraction of the background signal (PBS or medium only). Supplier unit definition was: one unit will produce 1.0 micromole of P-Nitroaniline from Gly-Pro-P-Nitroanilide per minute at pH 7.6 at 37°C. Plates were read in a Tristar LB941 device (Berthold Technologies, Oak Ridge, TN, USA). To account for potential effects on enzymatic activity in HIV infection, activity values were normalized by concentration, and both types of results are presented below.

**Quantification of IP-10**

The quantification of IP-10 (also known as CXCL10) was previously reported [44,48] and based on enzyme-linked immunosorbent assays. Briefly, the IP-10 concentrations were determined in stored plasma or sera samples (−80°C) using the commercially available Quantikine CXCL10 assay (R&D Systems, Minneapolis, Minnesota) according to the manufacturers' instruction.

**Quantification of gene expression**

Total RNA was extracted and reverse-transcribed as previously described [56]. qPCR (Taqman chemistry) and commercial kits were used to quantify the expression levels of genes of interest in the simian samples as previously reported [44]. Briefly, the Taqman primers and probes used were Hs00175210 for *DPP4*, Hs01076112_m1 for *RORc*, Hs00203436_m1 for *Tbx21* and 4310893^E^ for 18S. The expression of each gene was normalized to that of 18S rRNA, and relative expression levels were calculated using the ΔΔCT method by using as the internal reference the raw value for each gene in rectal CD4+ leukocytes from one rhesus macaque or, alternatively, by normalizing each value against the raw value of each gene in CD4+ leukocytes enriched from the rectum of each animal, as previously described [44].

The probes were initially designed for human sequences. We aligned the sequences that contain the primers and probes to the sequences from AGM and MAC (Supplementary Figure 1). The *DPP4* sequence was 100% identical between human, AGM and MAC. The Tbx21 sequences of AGM and MAC were 97.6% identical to the human sequence and 100% identical to each other. The RORc sequences were 100% identical in the region that covers the probe between the three species and 96.8%-98.4% identical in the remaining part. For the 18S sequence, the corresponding region is not available for AGM in the databases. The 18S MAC sequence was identical to the human one except for a 4 base pair insertion outside the region that covers the probe region. For 18S, we previously showed that the values obtained for AGM and MAC by RT-qPCR using 18SrRNA for normalization correlated to the values obtained by a distinct quantification method (microarrays) [57], indicating that the use of 18S rRNA for quantification of AGM and MAC mRNA gives robust results. Moreover, we can exclude that differences in the levels of *DPP4* and *Tbx21* mRNA as measured here are due to sequence differences. For *RORc* it is potentially possible but unlikely.

**Statistical analyses**

Continuous variables from the human cohorts were reported as medians and 25^th^ to 75^th^ percentiles (interquartile range, IQR). They were compared across groups with a Kruskall-Wallis test followed by Dunn’s test for multiple group comparisons, or a student t-test or Wilcoxon non-parametric tests for two-group comparisons, depending on the sample size. Categorical variables were compared across groups using the chi-2 test. Logistic regression was used to identify factors associated with rapid progression (RP, <350 CD4/mm^3^ by M12 post-seroconversion). In the ACS, time-to event analyses were used to analyze AIDS-related mortality and progression to CD4 levels <200 cells/mm^3^. For both outcomes, time-to-event was described using Kaplan-Meier non-parametric estimates and compared using the log-rank test. The Cox proportional hazard model was used to identify factors associated with the outcome. Correlations between markers were evaluated by calculating the Pearson correlation coefficient or Spearman’s correlation coefficient, depending on the normality of the data distribution and the group size. A P value <0.05 was considered statistically significant. All analyses were performed with Stata 13 software (StataCorp, College Station, Texas, USA) or GraphPad PRISM 6 (GraphPad software, La Jolla CA, USA). Plots and graphs were designed with GraphPad PRISM 6 (GraphPad software, La Jolla CA, USA).

**Results**

**Soluble plasma DPP4 is decreased in primary HIV infection**

It has been shown that circulating sDPP4 is reduced during chronic HIV infection [43]. If peripheral sDPP4 is a potential marker of intestinal Th17 loss, DPP4 loss would be rapid and already decreased during primary HIV-1 infection (PHI). We thus measured sDPP4 in the Amsterdam HIV Cohort (ACS) (Supplementary Table 1). The ACS disposes of a large collection of stored samples from individuals before and after HIV-1 infection. This cohort therefore enabled evaluation of sDPP4 levels in acute infection compared with the pre-infection period to document changes in response to HIV infection. In total, 136 patients were studied. sDPP4 was quantified before infection (N=87), in primary HIV infection (PHI, N=36), at 3 months post-seroconversion (M3, N=126) and 6 months post-seroconversion (M6, N=104). In most studies, the levels of sDDP4 are measured at the level of its enzymatic activity and absolute concentrations are more rarely presented [39,58–61]. We measured both and as in previous studies [62], found a strong correlation between the concentration and enzymatic activity of sDDP4 (R=0.72, p<0.0001 and supplementary Fig.2A). We thus performed the subsequent analyses with regard to the DPP4 activity levels. The levels of sDPP4 activity were statistically significantly lower during PHI and at M3 when compared to pre-infection (Fig.1A). Individuals for whom samples were available at all 4 time points (N=11), showed a similar profile (Fig.1B and Supplementary Fig.2B). At M6, the sDPP4 activity was higher than at M3, but in some cases still lower than pre-infection (Fig.1B and Supplementary Fig.2B).

We then examined if such a decrease of sDPP4 in PHI could also be observed in an independent cohort by quantifying sDPP4 in patients from the French ANRS PRIMO cohort [51]. The plasma samples of blood from 126 patients recruited in PHI and 87 healthy donors were analyzed. sDPP4 was statistically significantly lower in PHI when compared to healthy donors (Fig.1 C). The sDPP4 levels in PHI were lower than in the ACS cohort (Fig. 1A), but it cannot be excluded that this may be due to an earlier Fiebig stage in patients from the PRIMO cohort.

These data demonstrate that the levels of sDPP4 were already decreased in PHI. Then sDPP4 activity recovered progressively, but remained lowered compared to healthy donors.

**Soluble DPP4 levels were not restored under cART**

To study whether sDPP4 activity could be restored by cART, we quantified sDPP4 before and during cART in patients from the ANRS COPANA cohort, who were enrolled less than 6 months after diagnosis of HIV infection (Supplementary Table 1). The levels of sDPP4 in cART patients (N=71) were compared to those of viremic patients before cART (N=122), HIC (N=82) and healthy donors (N=87). Patients were on cART for >24 months, with effective viral control for >12 months (<50 copies of HIV RNA/mL).

The sDPP4 activity levels in the HIC were similar to those in healthy donors, but higher than in all other HIV groups, including the cART-treated individuals (Fig.1 C). Among the 82 HIC, 19 were HCV-infected. A high activity of sDPP4 was previously reported in HCV infection [38]. We compared the sDPP4 levels in the 19 HCV-infected HIC to those from the 63 HCV-negative HIC and observed no difference (sDPP4 IU/ml : p=0.18 ; sDPP4 ng/ml : p=0.34 ; sDPP4 IU/ng : p=0.55 ; Wilcoxon non parametric test). Furthermore, when we removed the 19 patients with HCV infection and analyzed the 63 remaining HCV-negative HIV controllers, the sDDP4 levels in the HIC were still higher than in all other HIV groups while not statistically significantly different from those in healthy donors (Supplementary Table 2.) Hence, we did not observe any impact of the HCV infection on the sDPP4 levels in the HIC studied here, and when comparisons were performed considering only HIC who are HCV-negative, the sDPP4 levels in the HIC were still higher than in all other HIV groups.

The sDPP4 activity levels in the viremic patients and cART patients were lower than those observed in healthy donors. Surprisingly, sDPP4 activity levels did not increase after cART (Fig.1 C). In order to further analyze this observation, we compared sDPP4 levels in 40 patients on cART for whom we also had a blood sample just before cART initiation. By comparing the same patients longitudinally, we observed a decrease of sDPP4 after cART initiation (Fig.1 D and supplementary Fig.2 C). The median fold change of sDPP4 before and after cART initiation was 0.77. Since cART regimens often comprise a protease inhibitor and because it is unknown whether the inhibitor can have an impact on the enzymatic activity of DPP4, we analyzed separately the individuals receiving a cART regimen with or without protease inhibitor. The decrease of sDPP4 remained statistically significant when considering the 20 patients that benefited from a combination of drugs containing a Protease inhibitor (median fold change 0.62), but not in patients that did not receive any PI (median fold change 0.86) as part of their therapeutic regimen (Fig.1 E, F and Supplementary Figures 2 D, E). Other parameters such as duration of infection were not different between the two groups.

The levels of sDPP4 levels were therefore not restored under cART and in some cases even further decreased.

**Low soluble DPP4 in primary HIV infection predicts rapid progression**

We next analyzed if the levels of sDPP4 during the early phase of the infection were associated with the disease progression profile. For this, the 126 patients from the PRIMO cohort were stratified into three groups, according to the kinetics of CD4^+^ T cell loss (slow progressors, typical progressors, rapid progressors), as described previously [48] and in the methods section. The enzymatic activity of sDPP4 in PHI was reduced most strongly in those patients who became rapid progressors, i.e. reached CD4^+^ T cell counts <350 within one year after PHI (Fig.2 A and Supplementary Fig.3).

Logistic regression analyses confirmed that patients with low levels of sDPP4 activity display an increased risk of becoming a rapid disease progressor (Table 1). We then analyzed sDPP4 compared to other markers predictive of disease progression, i.e. viremia, CD4^+^ T cell counts and IP-10. The latter has been identified as an early biomarker of rapid progression [48]. Multivariate analyses showed that sDPP4 was an independent marker of disease progression when including CD4 T cell levels, HIV RNA or DNA viral loads (Table 1). Of note, low levels of sDPP4 activity in PHI were better associated with rapid disease progression than viremia or cell-associated HIV DNA levels, although less than IP-10 (“co factor values”, Table 1).

Lastly, survival analyses were performed in the patients who were followed until clinical symptoms of AIDS and death (Amsterdam cohort). sDPP4 levels at M6, but not viremia, predicted the time until AIDS-related death (p=0.004) (Fig.2B).

In summary, we show that sDPP4 levels rapidly decrease during PHI, are not fully restored by cART initiated during chronic infection and predict disease progression.

***DPP4* expression profiles in the gut resemble those of RORC in intestinal CD4+ cells during SIV infection**

Since DPP4 has been described to be expressed at high levels in the gut [36], we next examined whether the reduction of these biomarker levels in the blood reflected changes in the gut epithelial barrier and the Th17 compartment. To do this, we took advantage of animal models to measure DPP4 expression in distinct compartments of the gut. We compared *DPP4* mRNA expression in the pathogenic model of HIV, i.e. rhesus macaques (MAC) infected by SIVmac to the non-pathogenic model, i.e. AGM infected by SIVagm [63]. AGM are known to preserve Th17 levels in the gut and to maintain an intact intestinal barrier despite high viremia [23]. The animals were infected with viral doses resulting in the same viremia levels in both species (as shown for instance in the supplementary Figure 1 of reference [64]). Gut biopsies were collected at day 65 post-infection (p.i.). Four distinct gut compartments were analyzed for each model corresponding to two compartments of the small intestine (ileum and jejunum) and two compartments from the large intestine (colon and rectum). Th17 cells are known to be most frequent in the small intestine [21,31,65]. To measure *in situ* production, we quantified the mRNA expression. *DPP4* mRNA was generally higher in the CD4^+^ cell fraction as compared to CD4^-^ cells for both MAC and AGM (Supplementary Fig.4A). In the AGM, DPP4 mRNA was higher in the small intestine than in the large intestine (Fig.3A). We then evaluated the correlation between plasma and intestinal *DPP4* in MAC and AGM. *DPP4* mRNA expression in the small intestine correlated with plasma DPP4 activity when values from the two distinct species were pooled (Fig.3B). Within each species, the correlations were not statistically significant, but we cannot exclude that the number of animals studied per species was too low to detect an association.

We next quantified *RORC* gene expression, a gene coding for the master transcription factor of Th17 cells, in the same samples (Fig.3C). *RORC* mRNA levels were higher in CD4^+^ cells of the small intestine than in those of the large intestine in AGM and higher in CD4+ than CD4- cells in the small intestine (Fig.3C and Suppl.Fig.4B). While *DPP4* and *RORC* gene expression profiles were similar to each other, *TBX21* mRNA showed a distinct profile from DPP4 and RORC (Fig.3E). *TBX21* mRNA was higher in CD4^+^ cells of the small than large intestine in MAC in contrast to *DPP4* and *RORC* that were not elevated. In line with this, *DPP4* and *RORC* mRNA expression in CD4^+^ cells positively correlated in the small intestine, while there was no correlation between *DPP4* and *TBX21* (Fig.3 D, F). *RORC* mRNA in the small intestine also positively correlated with plasma sDPP4 activity (R=0.67, p<0.04). As for *DPP4* mRNA, the correlations were only statistically significant when the results of the two species were pooled. We focused the analysis on the small intestine, because this is the major site for Th17 cells, but Th17 cells can also be detected in the rectum [28,31,66]. *DPP4* and *RORC* mRNA expression in CD4^+^ cells also positively correlated in the large intestine (R=0.79, p<0.05).

These results show that *DPP4* expressions were highest in the CD4^+^ cell fraction from the small intestine in AGM. The *DPP4* gene expression profiles were distinct from that of *TBX21* and rather resembled those of *RORC*. One possible explanation is that RORC^+^CD4^+^ cells, corresponding to Th17 cells, contribute to DPP4 production in the gut.

**sDPP4 levels are increased in animals treated with IL-21**

To further investigate links between sDPP4 blood levels and intestinal Th17 cells, we tested the hypothesis that animals showing increases in intestinal Th17 cells subsequent to an immunotherapy would also display increases in peripheral sDPP4. Pre-clinical studies in the macaque model have shown promising results with IL-21 treatment regarding the restoration of intestinal Th17 cells [28,31,66]. We therefore quantified sDPP4 in plasma samples from SIV-infected macaques that had been treated with IL-21. These animals had received weekly rIL-21 from week two p.i. for four weeks [31]. Six macaques treated with IL-21 and 6 control animals were analyzed (Fig.4A). Peripheral sDPP4 levels indeed increased in the IL-21 treated animals. The animals showed a trend for sDPP4 increase at the end of the IL-21 treatment (week 6 p.i) and a statistically significant increase at the following time point that was 4 weeks after IL-21 administration (week 10 p.i, *p* < 0.01).

It has been previously described that the increase of Th17 cells in the gut of these animals were the strongest by the end of IL-21 therapy, i.e. at week 4-6 p.i. [31]. Th17 cells can be further categorized into Th17 (IL17^+^) and Th1Th17 (IFN-γ^+^IL17^+^) cells. Since Th17 cells have been described to express the highest levels of DPP4 among CD4^+^ T cells [36], both cell populations (IL17^+^ and IFN-γ^++^IL17^+^ CD4 T cells in the gut) were analyzed. The percentages of IL17+ and IFN-γ^+^IL17^+^ CD4^+^ T cells in the IL-21 treated animals were previously measured and published [31] and these values were used here to compare the correlations between IL-17^+^ cells and sDPP4 levels at time points where the changes of IL-17^+^ cells were reported to be statistically significant (week 4 and week 6). The frequencies of intestinal IL-17^+^CD4^+^ and IL-17^+^IFN-g^+^CD4^+^ cells at these time points correlated with the subsequent increased levels of sDPP4 (Fig.4 B, C).

Also, the previous study showed that immune activation and microbial translocation markers were markedly decreased at Week 23 (end of the study) in IL21-treated animals [31]. We used the previously [31] determined levels of these markers (intestinal Ki67^+^CD4^+^T cells, plasma sCD14, plasma LPS and plasma IP-10) and analysed the correlation with sDPP4 levels. Interestingly, sDPP4 levels negatively correlated with immune activation markers (CD4^+^ T cell proliferation in the gut, plasma IP-10 and plasma LPS, Fig. 4D, E, G). There also was a trend for a negative correlation with sCD14 but this was not statistically significant (Fig. 4F).

Together, these data indicate that IL-21 therapy increased systemic sDPP4 activity. This sDPP4 increase could be indirect and a consequence of Th17 cell increases in the gut.

**Discussion**

DPP4 levels have been studied in many non-communicable pathologies, and abnormal levels have been observed in metabolic disorders (i.e. type II diabetes and metabolic syndrome) [67,68], auto-immunity (i.e. multiple sclerosis, rheumatoid arthritis) [59,69] and cancer (i.e. B-cell leukemia, hepatocellular carcinoma, bladder cancer) [60,70,71]. In addition, DPP4 inhibitors are a widely used therapy for type II diabetes [72]. In the setting of cancer, it has recently been proposed that DPP4 inhibitors could enhance trafficking of anti-tumor immune cells into the lesions [73]. DPP4 has been less studied in viral infections, with the exception of HCV [38,74]. A study on HIV infection provided evidence that immunomodulation by Ursodeoxycholic acid can increase the number of CD4^+^DPP4^+^ T cells in AIDS patients [61]. Herein, we have studied for the first time sDPP4 levels in primary HIV infection, demonstrating that sDPP4 is severely decreased during PHI in two independent cohorts. sDPP4 levels were also lower in chronic HIV infection, as compared to healthy controls. This is in line with previous studies reporting decreased levels of sDPP4 in chronic HIV infection [43]. Furthermore we also report here DPP4 levels through a longitudinal analysis, starting prior to initial infection and revealing that the sDPP4 levels recovered only partially after PHI, and remained lower than in healthy donors.

Our study also reveals a strong association between plasma sDPP4 activity and disease progression. Thus, sDPP4 activity was most reduced during PHI in HIV-infected patients who subsequently progressed rapidly. This was validated in two independent cohorts. Of note, the sDPP4 levels in PHI had a stronger predictive value for disease progression than viral load in PHI. However, as the difference in sDPP4 levels between rapid progressors and slow/normal progressors, although statistically significant, was small, measuring sDDP4 seems unlikely to be a useful measure in clinics of rapid disease progression in newly infected patients. We previously reported that plasma IP-10 levels are increased in PHI, especially in rapid progressors [48]. During PHI, IP-10 levels were even more predictive of disease progression than RNA viremia and CD4 T-cell counts, while soluble CD163 levels were not. Here we analyzed the same patients as in the previous studies [44,48]. Remarkably, sDPP4 levels in PHI were also more predictive than CD4 T cell counts, viremia and total cell-associated HIV DNA for rapid disease progression. However, the predictive values were lower than those for IP-10. IP-10 thus seems to be a better early marker for rapid disease progression than sDPP4.

Our animal studies allowed us to analyze DPP4 expression in the gut. *DPP4* mRNA expression was higher in the CD4+ than the CD4- fraction. Th17 cells have been described to express the highest levels of DPP4 among CD4^+^ T cells [36], but DPP4 is known to be also expressed by other intestinal cells, such as MAIT cells [75]. We cannot exclude that the decrease of sDPP4 that we observed in the blood of the patients and the gut of the macaques was solely due to the loss of Th17 cells. However, *DPP4* mRNA expression was associated with *RORC* mRNA expression in CD4+ cells from the gut, supporting that the loss of DPP4 is at least partially related to dynamics of mucosal Th17 cells.

Studies with HIV-1 exposed but non-infected female sex workers revealed that their resistance to HIV infection was associated with increased concentrations of blood sDPP4 and elevated sDPP4 expression (gene and protein level) in PBMCs [76,77]. The reasons for this are not well understood. In this context though it is interesting to note that SIV replication in the macaque is limited by the size of the preexisting Th17 cell compartment [78]. Indeed, in that study it was shown that animals with a high level of Th17 cells in intestinal tissue before infection experienced lower viral load. DPP4 was not measured in that study and it is theoretically possible that the higher DPP4 levels in the exposed but non infected individuals reflected higher intestinal Th17 levels. Our studies in IL-21 treated, SIV-infected animals further support the link between sDPP4 in blood and intestinal Th17 levels. Here, DPP4 levels were increased after IL-21 therapy in the animal model and correlated with increases in intestinal Th17 cells.

It was striking to observe that in patients under cART, the sDPP4 activity levels did not increase. This contrasts with a study reporting that cART leads to a gain of plasma sDPP4 activity [43]. This study however included only 2 patients, none of whom received a protease inhibitor. Future studies are needed to evaluate whether anti-HIV protease inhibitors influence sDPP4 activity, as compared with other antiretroviral regimen such as integrase inhibitors. In case anti-HIV protease inhibitors would impact DPP4 activity, we cannot exclude that residual protease inhibitors within the plasma samples were affecting the enzyme activity assay. This has implications if sDDP4 is to be used as a surrogate marker to determine if new therapies are improving the recovery of the gastrointestinal tract in individuals receiving PI-cART. In the patients here, cART was initiated only in the chronic phase and relatively recently (median 34 months) [46,47]. It is thus possible that sDPP4 levels did not increase because cART in these chronically HIV-infected, recently treated patients did not restore gut Th17 cells.

Soluble DPP4 is already an established biomarker in other diseases such as type II diabetes [67,68]. We propose that sDPP4 could be used as a systemic surrogate marker for evaluation of the capacity of potential therapies to restore gut immune function, in particular Th17 levels in the gut during HIV infection. A curative strategy of HIV will likely need to both reduce the amount of virus that persists on antiretroviral therapy and improve anti-HIV immune surveillance [15,79]. Altogether, our study reveals the potential benefit of using sDPP4 as a biomarker in the field of HIV cure/remission and paves the way for future studies aimed at evaluating its potential use as a biomarker for restoration of gut immunity.

**ACKNOWLEDGMENTS**

We deeply thank the patients and healthy donors. We are grateful to the physicians at the hospitals. We thank all members of the steering committees of the ANRS C06, C09 and C021 cohorts and those of the ACS. We are grateful to the excellent work by the veterinarians, animal care workers and the staff of the IDMIT animal facilities, in particular Nathalie Derreudre-Bosquet, Jean-Marie Helies, Benoit Delache and Christophe Joubert. We would like to acknowledge strongly the Centre d'Immunologie Humaine (CIH) at Institut Pasteur as well as the IDMIT Center at the Commissariat à l’Energie Atomique et aux energies alternatives (CEA) for their state-of-the-art equipment and core facilities.

**References**

1. Maartens G, Celum C, Lewin SR. HIV infection: epidemiology, pathogenesis, treatment, and prevention. Lancet 2014;384:258–71.

2. Barré-Sinoussi F, Ross AL, Delfraissy J-F. Past, present and future: 30 years of HIV research. Nat. Rev. Microbiol. 2013;11:877–83.

3. Katlama C, Deeks SG, Autran B, Martinez-Picado J, van Lunzen J, Rouzioux C, et al. Barriers to a cure for HIV: new ways to target and eradicate HIV-1 reservoirs. Lancet Lond. Engl. 2013;381:2109–17.

4. Churchill MJ, Deeks SG, Margolis DM, Siliciano RF, Swanstrom R. HIV reservoirs: what, where and how to target them. Nat. Rev. Microbiol. 2016;14:55–60.

5. Deleage C, Wietgrefe SW, Del Prete G, Morcock DR, Hao XP, Piatak M, et al. Defining HIV and SIV Reservoirs in Lymphoid Tissues. Pathog. Immun. 2016;1:68–106.

6. Sereti I, Krebs SJ, Phanuphak N, Fletcher JL, Slike B, Pinyakorn S, et al. Persistent, Albeit Reduced, Chronic Inflammation in Persons Starting Antiretroviral Therapy in Acute HIV Infection. Clin. Infect. Dis. 2017;64:124–31.

7. Boulware DR, Hullsiek KH, Puronen CE, Rupert A, Baker JV, French MA, et al. Higher levels of CRP, D-dimer, IL-6, and hyaluronic acid before initiation of antiretroviral therapy (ART) are associated with increased risk of AIDS or death. J. Infect. Dis. 2011;203:1637–46.

8. French MA, King MS, Tschampa JM, da Silva BA, Landay AL. Serum immune activation markers are persistently increased in patients with HIV infection after 6 years of antiretroviral therapy despite suppression of viral replication and reconstitution of CD4+ T cells. J. Infect. Dis. 2009;200:1212–5.

9. Hunt PW, Brenchley J, Sinclair E, McCune JM, Roland M, Page-Shafer K, et al. Relationship between T cell activation and CD4+ T cell count in HIV-seropositive individuals with undetectable plasma HIV RNA levels in the absence of therapy. J. Infect. Dis. 2008;197:126–33.

10. Noel N, Boufassa F, Lécuroux C, Saez-Cirion A, Bourgeois C, Dunyach-Remy C, et al. Elevated IP10 levels are associated with immune activation and low CD4^+^ T-cell counts in HIV controller patients. AIDS 2014;28:467–76.

11. Krishnan S, Wilson EMP, Sheikh V, Rupert A, Mendoza D, Yang J, et al. Evidence for innate immune system activation in HIV type 1-infected elite controllers. J. Infect. Dis. 2014;209:931–9.

12. Borges ÁH, Silverberg MJ, Wentworth D, Grulich AE, Fätkenheuer G, Mitsuyasu R, et al. Predicting risk of cancer during HIV infection: the role of inflammatory and coagulation biomarkers. AIDS 2013;27:1433–41.

13. Nordell AD, McKenna M, Borges ÁH, Duprez D, Neuhaus J, Neaton JD, et al. Severity of cardiovascular disease outcomes among patients with HIV is related to markers of inflammation and coagulation. J. Am. Heart Assoc. 2014;3:e000844.

14. Siliciano JD, Siliciano RF. Recent developments in the effort to cure HIV infection: going beyond N = 1. J. Clin. Invest. 2016;126:409–14.

15. Lewin SR, Deeks SG, Barré-Sinoussi F. Towards a cure for HIV--are we making progress? Lancet 2014;384:209–11.

16. Paiardini M, Müller-Trutwin M. HIV-associated chronic immune activation. Immunol. Rev. 2013;254:78–101.

17. Zevin AS, McKinnon L, Burgener A, Klatt NR. Microbial translocation and microbiome dysbiosis in HIV-associated immune activation. Curr. Opin. HIV AIDS 2016;11:182–90.

18. Brenchley JM, Price DA, Schacker TW, Asher TE, Silvestri G, Rao S, et al. Microbial translocation is a cause of systemic immune activation in chronic HIV infection. Nat. Med. 2006;12:1365–71.

19. Schuetz A, Deleage C, Sereti I, Rerknimitr R, Phanuphak N, Phuang-Ngern Y, et al. Initiation of ART during early acute HIV infection preserves mucosal Th17 function and reverses HIV-related immune activation. PLoS Pathog. 2014;10:e1004543.

20. Deleage C, Schuetz A, Alvord WG, Johnston L, Hao X-P, Morcock DR, et al. Impact of early cART in the gut during acute HIV infection. JCI Insight 2016;1.

21. Mudd JC, Brenchley JM. Gut Mucosal Barrier Dysfunction, Microbial Dysbiosis, and Their Role in HIV-1 Disease Progression. J. Infect. Dis. 2016;214 Suppl 2:S58-66.

22. Somsouk M, Dunham RM, Cohen M, Albright R, Abdel-Mohsen M, Liegler T, et al. The immunologic effects of mesalamine in treated HIV-infected individuals with incomplete CD4+ T cell recovery: a randomized crossover trial. PloS One 2014;9:e116306.

23. Kristoff J, Haret-Richter G, Ma D, Ribeiro RM, Xu C, Cornell E, et al. Early microbial translocation blockade reduces SIV-mediated inflammation and viral replication. J. Clin. Invest. 2014;124:2802–6.

24. Sandler NG, Zhang X, Bosch RJ, Funderburg NT, Choi AI, Robinson JK, et al. Sevelamer does not decrease lipopolysaccharide or soluble CD14 levels but decreases soluble tissue factor, low-density lipoprotein (LDL) cholesterol, and oxidized LDL cholesterol levels in individuals with untreated HIV infection. J. Infect. Dis. 2014;210:1549–54.

25. Tenorio AR, Chan ES, Bosch RJ, Macatangay BJC, Read SW, Yesmin S, et al. Rifaximin has a marginal impact on microbial translocation, T-cell activation and inflammation in HIV-positive immune non-responders to antiretroviral therapy - ACTG A5286. J. Infect. Dis. 2015;211:780–90.

26. Manuzak JA, Hensley-McBain T, Zevin AS, Miller C, Cubas R, Agricola B, et al. Enhancement of Microbiota in Healthy Macaques Results in Beneficial Modulation of Mucosal and Systemic Immune Function. J. Immunol. 2016;196:2401–9.

27. Hensley-McBain T, Zevin AS, Manuzak J, Smith E, Gile J, Miller C, et al. Effects of Fecal Microbial Transplantation on Microbiome and Immunity in Simian Immunodeficiency Virus-Infected Macaques. J. Virol. 2016;90:4981–9.

28. Ortiz AM, Klase ZA, DiNapoli SR, Vujkovic-Cvijin I, Carmack K, Perkins MR, et al. IL-21 and probiotic therapy improve Th17 frequencies, microbial translocation, and microbiome in ARV-treated, SIV-infected macaques. Mucosal Immunol. 2016;9:458–67.

29. d’Ettorre G, Ceccarelli G, Giustini N, Serafino S, Calantone N, De Girolamo G, et al. Probiotics Reduce Inflammation in Antiretroviral Treated, HIV-Infected Individuals: Results of the “Probio-HIV” Clinical Trial. PloS One 2015;10:e0137200.

30. Klatt NR, Canary LA, Sun X, Vinton CL, Funderburg NT, Morcock DR, et al. Probiotic/prebiotic supplementation of antiretrovirals improves gastrointestinal immunity in SIV-infected macaques. J. Clin. Invest. 2013;123:903–7.

31. Pallikkuth S, Micci L, Ende ZS, Iriele RI, Cervasi B, Lawson B, et al. Maintenance of intestinal Th17 cells and reduced microbial translocation in SIV-infected rhesus macaques treated with interleukin (IL)-21. PLoS Pathog. 2013;9:e1003471.

32. Chevalier MF, Petitjean G, Dunyach-Rémy C, Didier C, Girard P-M, Manea ME, et al. The Th17/Treg ratio, IL-1RA and sCD14 levels in primary HIV infection predict the T-cell activation set point in the absence of systemic microbial translocation. PLoS Pathog. 2013;9:e1003453.

33. Marchetti G, Cozzi-Lepri A, Merlini E, Bellistrì GM, Castagna A, Galli M, et al. Microbial translocation predicts disease progression of HIV-infected antiretroviral-naive patients with high CD4+ cell count. AIDS 2011;25:1385–94.

34. León A, Leal L, Torres B, Lucero C, Inciarte A, Arnedo M, et al. Association of microbial translocation biomarkers with clinical outcome in controllers HIV-infected patients. AIDS 2015;29:675–81.

35. Abad-Fernández M, Vallejo A, Hernández-Novoa B, Díaz L, Gutiérrez C, Madrid N, et al. Correlation between different methods to measure microbial translocation and its association with immune activation in long-term suppressed HIV-1-infected individuals. J. Acquir. Immune Defic. Syndr. 1999 2013;64:149–53.

36. Bengsch B, Seigel B, Flecken T, Wolanski J, Blum HE, Thimme R. Human Th17 cells express high levels of enzymatically active dipeptidylpeptidase IV (CD26). J. Immunol. 2012;188:5438–47.

37. Ohnuma K, Dang NH, Morimoto C. Revisiting an old acquaintance: CD26 and its molecular mechanisms in T cell function. Trends Immunol. 2008;29:295–301.

38. Casrouge A, Decalf J, Ahloulay M, Lababidi C, Mansour H, Vallet-Pichard A, et al. Evidence for an antagonist form of the chemokine CXCL10 in patients chronically infected with HCV. J. Clin. Invest. 2011;121:308–17.

39. De Pasquale A, Ginaldi L, Limoncelli P, Quaglino D. Dipeptidyl amino peptidase IV cytochemistry in circulating lymphocytes from HIV-I-seropositive subjects. Acta Haematol. 1989;81:19–21.

40. Blazquez MV, Madueño JA, Gonzalez R, Jurado R, Bachovchin WW, Peña J, et al. Selective decrease of CD26 expression in T cells from HIV-1-infected individuals. J. Immunol.1992;149:3073–7.

41. Vanham G, Kestens L, De Meester I, Vingerhoets J, Penne G, Vanhoof G, et al. Decreased expression of the memory marker CD26 on both CD4+ and CD8+ T lymphocytes of HIV-infected subjects. J. Acquir. Immune Defic. Syndr. 1993;6:749–57.

42. Gougeon ML, Lecoeur H, Callebaut C, Jacotot E, Dulioust A, Roué R, et al. Selective loss of the CD4+/CD26+ T-cell subset during HIV infection. Res. Immunol. 1996;147:5–8.

43. Hosono O, Homma T, Kobayashi H, Munakata Y, Nojima Y, Iwamoto A, et al. Decreased dipeptidyl peptidase IV enzyme activity of plasma soluble CD26 and its inverse correlation with HIV-1 RNA in HIV-1 infected individuals. Clin. Immunol. 1999;91:283–95.

44. Ploquin MJ, Madec Y, Casrouge A, Huot N, Passaes C, Lécuroux C, et al. Elevated Basal Pre-infection CXCL10 in Plasma and in the Small Intestine after Infection Are Associated with More Rapid HIV/SIV Disease Onset. PLoS Pathog. 2016;12:e1005774.

45. Euler Z, van Gils MJ, Boeser-Nunnink BD, Schuitemaker H, van Manen D. Genome-wide association study on the development of cross-reactive neutralizing antibodies in HIV-1 infected individuals. PloS One 2013;8:e54684.

46. Boufassa F, Goujard C, Viard J-P, Carlier R, Lefebvre B, Yeni P, et al. Immune deficiency could be an early risk factor for altered insulin sensitivity in antiretroviral-naive HIV-1-infected patients: the ANRS COPANA cohort. Antivir. Ther. 2012;17:91–100.

47. Ghislain M, Bastard J-P, Meyer L, Capeau J, Fellahi S, Gérard L, et al. Late Antiretroviral Therapy (ART) Initiation Is Associated with Long-Term Persistence of Systemic Inflammation and Metabolic Abnormalities. PloS One 2015;10:e0144317.

48. Liovat A-S, Rey-Cuillé M-A, Lécuroux C, Jacquelin B, Girault I, Petitjean G, et al. Acute plasma biomarkers of T cell activation set-point levels and of disease progression in HIV-1 infection. PloS One 2012;7:e46143.

49. Ghosn J, Pellegrin I, Goujard C, Deveau C, Viard J-P, Galimand J, et al. HIV-1 resistant strains acquired at the time of primary infection massively fuel the cellular reservoir and persist for lengthy periods of time. AIDS 2006;20:159–70.

50. Goujard C, Bonarek M, Meyer L, Bonnet F, Chaix M-L, Deveau C, et al. CD4 cell count and HIV DNA level are independent predictors of disease progression after primary HIV type 1 infection in untreated patients. Clin. Infect. Dis. 2006;42:709–15.

51. Laanani M, Ghosn J, Essat A, Melard A, Seng R, Gousset M, et al. Impact of the Timing of Initiation of Antiretroviral Therapy During Primary HIV-1 Infection on the Decay of Cell-Associated HIV-DNA. Clin. Infect. Dis. 2015;60:1715–21.

52. Ngo-Giang-Huong N, Deveau C, Da Silva I, Pellegrin I, Venet A, Harzic M, et al. Proviral HIV-1 DNA in subjects followed since primary HIV-1 infection who suppress plasma viral load after one year of highly active antiretroviral therapy. AIDS 2001;15:665–73.

53. Diop OM, Gueye A, Dias-Tavares M, Kornfeld C, Faye A, Ave P, et al. High levels of viral replication during primary simian immunodeficiency virus SIVagm infection are rapidly and strongly controlled in African green monkeys. J. Virol. 2000;74:7538–47.

54. Jacquelin B, Petitjean G, Kunkel D, Liovat A-S, Jochems SP, Rogers KA, et al. Innate immune responses and rapid control of inflammation in African green monkeys treated or not with interferon-alpha during primary SIVagm infection. PLoS Pathog. 2014;10:e1004241.

55. Pandrea I, Kornfeld C, Ploquin MJ-Y, Apetrei C, Faye A, Rouquet P, et al. Impact of viral factors on very early in vivo replication profiles in simian immunodeficiency virus SIVagm-infected African green monkeys. J. Virol. 2005;79:6249–59.

56. Kornfeld C, Ploquin MJ-Y, Pandrea I, Faye A, Onanga R, Apetrei C, et al. Antiinflammatory profiles during primary SIV infection in African green monkeys are associated with protection against AIDS. J. Clin. Invest. 2005;115:1082–91.

57. Jacquelin B, Mayau V, Targat B, Liovat A-S, Kunkel D, Petitjean G, et al. Nonpathogenic SIV infection of African green monkeys induces a strong but rapidly controlled type I IFN response. J. Clin. Invest. 2009;119:3544–55.

58. Söderholm J, Waldenström J, Askarieh G, Pilli M, Bochud P-Y, Negro F, et al. Impact of soluble CD26 on treatment outcome and hepatitis C virus-specific T cells in chronic hepatitis C virus genotype 1 infection. PloS One 2013;8:e56991.

59. Tejera-Alhambra M, Casrouge A, de Andrés C, Ramos-Medina R, Alonso B, Vega J, et al. Low DPP4 expression and activity in multiple sclerosis. Clin. Immunol. 2014;150:170–83.

60. Liang P-I, Yeh B-W, Li W-M, Chan T-C, Chang I-W, Huang C-N, et al. DPP4/CD26 overexpression in urothelial carcinoma confers an independent prognostic impact and correlates with intrinsic biological aggressiveness. Oncotarget 2017;8:2995–3008.

61. Kurktschiev D, Temelkova-Kurktschiev T, Horn K, Schentke K. Successful immunomodulating in AIDS patients with ursodeoxycholic acid--a pilot study. Clin. Exp. Immunol. 1999;115:144–6.

62. Meissner EG, Decalf J, Casrouge A, Masur H, Kottilil S, Albert ML, et al. Dynamic Changes of Post-Translationally Modified Forms of CXCL10 and Soluble DPP4 in HCV Subjects Receiving Interferon-Free Therapy. PloS One 2015;10:e0133236.

63. Garcia-Tellez T, Huot N, Ploquin MJ, Rascle P, Jacquelin B, Müller-Trutwin M. Non-human primates in HIV research: Achievements, limits and alternatives. Infect. Genet. Evol. 2016;46:324–32.

64. Huot N, Jacquelin B, Garcia-Tellez T, Rascle P, Ploquin MJ, Madec Y, et al. Natural killer cells migrate into and control simian immunodeficiency virus replication in lymph node follicles in African green monkeys. Nat. Med. 2017;23:1277–86.

65. Mowat AM, Agace WW. Regional specialization within the intestinal immune system. Nat. Rev. Immunol. 2014;14:667–85.

66. Micci L, Ryan ES, Fromentin R, Bosinger SE, Harper JL, He T, et al. Interleukin-21 combined with ART reduces inflammation and viral reservoir in SIV-infected macaques. J. Clin. Invest. 2015;125:4497–513.

67. Lee SA, Kim YR, Yang EJ, Kwon E-J, Kim SH, Kang SH, et al. CD26/DPP4 levels in peripheral blood and T cells in patients with type 2 diabetes mellitus. J. Clin. Endocrinol. Metab. 2013;98:2553–61.

68. Zheng T, Gao Y, Baskota A, Chen T, Ran X, Tian H. Increased plasma DPP4 activity is predictive of prediabetes and type 2 diabetes onset in Chinese over a four-year period: result from the China National Diabetes and Metabolic Disorders Study. J. Clin. Endocrinol. Metab. 2014;99:E2330-2334.

69. Grujic M, Matic IZ, Crnogorac MD, Velickovic AD, Kolundzija B, Cordero OJ, et al. Activity and expression of dipeptidyl peptidase IV on peripheral blood mononuclear cells in patients with early steroid and disease modifying antirheumatic drugs naïve rheumatoid arthritis. Clin. Chem. Lab. Med. 2017;55:73–81.

70. Stecca BA, Nardo B, Chieco P, Mazziotti A, Bolondi L, Cavallari A. Aberrant dipeptidyl peptidase IV (DPP IV/CD26) expression in human hepatocellular carcinoma. J. Hepatol. 1997;27:337–45.

71. Molica S, Digiesi G, Mirabelli R, Cutrona G, Antenucci A, Molica M, et al. Serum level of CD26 predicts time to first treatment in early B-chronic lymphocytic leukemia. Eur. J. Haematol. 2009;83:208–14.

72. Drucker DJ, Nauck MA. The incretin system: glucagon-like peptide-1 receptor agonists and dipeptidyl peptidase-4 inhibitors in type 2 diabetes. Lancet 2006;368:1696–705.

73. Barreira da Silva R, Laird ME, Yatim N, Fiette L, Ingersoll MA, Albert ML. Dipeptidylpeptidase 4 inhibition enhances lymphocyte trafficking, improving both naturally occurring tumor immunity and immunotherapy. Nat. Immunol. 2015;16:850–8.

74. Ragab D, Laird M, Duffy D, Casrouge A, Mamdouh R, Abass A, et al. CXCL10 antagonism and plasma sDPPIV correlate with increasing liver disease in chronic HCV genotype 4 infected patients. Cytokine 2013;63:105–12.

75. Sharma PK, Wong EB, Napier RJ, Bishai WR, Ndung’u T, Kasprowicz VO, et al. High expression of CD26 accurately identifies human bacteria-reactive MR1-restricted MAIT cells. Immunology 2015;145:443–53.

76. Songok EM, Osero B, McKinnon L, Rono MK, Apidi W, Matey EJ, et al. CD26/dipeptidyl peptidase IV (CD26/DPPIV) is highly expressed in peripheral blood of HIV-1 exposed uninfected female sex workers. Virol. J. 2010;7:343.

77. Songok EM, Luo M, Liang B, Mclaren P, Kaefer N, Apidi W, et al. Microarray analysis of HIV resistant female sex workers reveal a gene expression signature pattern reminiscent of a lowered immune activation state. PloS One 2012;7:e30048.

78. Hartigan-O’Connor DJ, Abel K, Van Rompay KKA, Kanwar B, McCune JM. SIV replication in the infected rhesus macaque is limited by the size of the preexisting TH17 cell compartment. Sci. Transl. Med. 2012;4:136ra69.

79. Saez-Cirion A, Jacquelin B, Barré-Sinoussi F, Müller-Trutwin M. Immune responses during spontaneous control of HIV and AIDS: what is the hope for a cure? Philos. Trans. R. Soc. Lond. B. Biol. Sci. 2014;369:20130436.

80. Mellors JW, Rinaldo CR, Gupta P, White RM, Todd JA, Kingsley LA. Prognosis in HIV-1 infection predicted by the quantity of virus in plasma. Science 1996;272:1167–70.

**Supporting information**

Supplementary Figures 1-4

Supplementary Tables 1

**Funding statement**

This work was supported by the ANRS and the Fondation l’Oréal. MJP was the recipient of a SIDACTION Young Investigator fellowship. SPJ received a PhD fellowship from the French Ministry of Higher Education and Research and TGT from the Pasteur Paris University International PhD program and Institut Carnot Microbes et Santé. NH received a postdoctoral fellowship from the French Vaccine Research Institute (Créteil, France), which is supported by the « Investissements d’Avenir program » managed by the National Agency of Research (ANR) under reference ANR-10-LABX-77. The Center for Infectious Disease models and Innovative Therapies (IDMIT) is supported by the French government “Programme d’Investissements d’Avenir” (PIA) under Grant ANR-11-INBS-0008 funding the Infectious Disease Models and Innovative Therapies (IDMIT, Fontenay-aux-Roses, France) infrastructure, the PIA grant ANR-10-EQPX-02-01 funding the FlowCyTech facility (IDMIT, Fontenay-aux-Roses, France). This work was supported by the ANRS and also by the NIH under award numbers AI116379 (to MP) and by ORIP/OD P51OD011132 (formerly NCRR P51RR000165, to the YNPRC). The funders had no role in the study design, data collection and analysis, decision to publish, or preparation of the manuscript.

### Competing interests

The authors declare that no competing interests exist.

**Authors contributions**.

MJP and AC performed the ELISA experiments. MJP performed the qPCR assays. MJP, BJ, NH, SJP and TGT performed the intestinal tissue preparations. CL, MG, OL, CG and NK provided human samples. RLG, LM and MP provided simian samples. FB, TB, MG, NK and LM performed data curation regarding samples from the human cohorts. AC and DD developed tools. MJP, DD, MA and MMT developed the concept of the study and its experimental design. All authors participated in the analyses and interpretation of the data. MJP, YM and NN performed the statistical analyses and LM supervised them. MJP, NN and YM prepared the figures. BJ wrote the animal protocols. MMT wrote the human protocols, supervised the research and wrote the manuscript, which was reviewed by the co-authors.

**Table 1: Logistic regression for evaluation of the capacity of sDPP4 in PHI compared with other markers to predict disease progression.**

|  | sDPP4 | Adj. OR  (95% CI) | p | Co-factor | Adj. OR  (95% CI) | p |
| --- | --- | --- | --- | --- | --- | --- |
| Absolute level of  sDPP4 | ≤4.18 >4.18 | 4.56 (1.72-12.12)  1 | **0.002** | IP10  ≤233  >233 | 1  9.78 (3.32-28.75) | **<0.001** |
| activity  (IU/mL) | ≤4.18 >4.18 | 4.98 (1.81-13.71)  1 | **0.002** | CD4, cells/mm^3^ *  ≤350  351-500  501-750  >750 | 7.45 (1.14-48.54)  0.74 (0.26-2.10)  1  0.22 (0.05-1.09) | **0.026** |
|  | ≤4.18 >4.18 | 4.32 (1.66-11.23)  1 | **0.003** | CD4, cells/mm^3^ *  ≤500  501-750  >750 | 1.19 (0.47-2.98)  1  0.22 (0.05-1.07) | 0.12 |
|  | ≤4.18 >4.18 | 4.13 (1.64-10.38)  1 | **0.003** | vRNA, log cp/mL  ≤4.00  4.01-5.00  >5.00 | 1  6.67 (0.75-56.95)  9.30 (1.13-76.76) | 0.11 |
|  | ≤4.18 >4.18 | 3.80 (1.51-9.59)  1 | **0.005** | ca-DNA, log cp/10^6^ PBMC**  ≤2.85  >2.85 | 1  2.37 (0.72-7.78) | 0.16 |
| Norma-lized  level of | ≤0.010 >0.010 | 3.79 (1.48 – 9.71) 1 | **0.006** | IP10  ≤233  >233 | 1 10.27 (3.5-30.1) | **<0.001** |
| sDPP4 activity  (IU/ng) | ≤0.010 >0.010 | 2.66 (1.08-6.53)  1 | **0.033** | CD4, cells/mm^3^ *  ≤350  351-500  501-750  >750 | 4.41 (0.75-26.07)  0.81 (0.30-2.24)  1  0.19 (0.04-0.89) | **0.043** |
|  | ≤0.010 >0.010 | 2.93  (1.21-7.08)  1 | **0.017** | CD4, cells/mm^3^ *  ≤500  501-750  >750 | 1.15 (0.46-2.87)  1  0.19 (0.04-0.89) | 0.078 |
|  | ≤0.010 >0.010 | 3.54  (1.48-8.52)  1 | **0.005** | vRNA, log cp/mL  ≤4.00  4.01-5.00  >5.00 | 1  6.64 (0.79-56.23)  11.43 (1.4-93.98) | 0.055 |
|  | ≤0.010 >0.010 | 2.87  (1.20-6.87)  1 | **0.018** | ca-DNA, log cp/10^6^ PBMC **  ≤2.85  >2.85 | 1  2.95 (0.92-9.46) | 0.069 |

Logistic regression was performed to evaluate the capacity of DPP4 compared to other markers to predict disease progression by bivariate analysis. We analyzed both the absolute levels of sDPP4 enzymatic activity as well as the levels of normalized sDPP4 activity. The analyses were performed on data from the 126 patients of the PRIMO cohort. The viral load and IP-10 levels were determined in [48]. If less samples were available, this is indicated in the table: * 125 patients, ** 117 patients. All values were from PHI. The thresholds for DPP4 and IP10 were based on the median; for the CD4 count they correspond to historical thresholds for antiretroviral treatment initiation (350 and 500 cells/mm3) and an arbitrary threshold at 750 cells/mm3 was also used as the distribution was off-centered towards larger values. For plasma viral RNA we used thresholds at 4 and 5 log copies/mL as these are cutoffs usually used in the literature in pathogenesis studies to separate progressors from long-term non progressors [80]; for DNA we used 25^th^ percentile to gain statistical power as the univariate analysis in 4 categories showed an effect below the threshold. vRNA: plasma HIV RNA; ca-DNA: cell (PBMC)-associated viral DNA. Adj. OR: adjusted Odds Ratio. CI: confidence interval; cp: copies.

**FIGURE LEGENDS**

**Figure 1. Soluble DPP4 levels in blood over time during distinct phases of HIV-1 infection. (A)** Soluble DPP4 activity levels over time in patients from the Amsterdam cohort Study (ACS). Blood collected before HIV-1 infection and at early time points post-infection were analyzed. (**B)**. Soluble DPP4 activity levels in 11 of these patients from the ACS for whom samples at all four time points of the study were available. **(C)** Soluble DPP4 activity levels in healthy donors (HD), in HIV-infected treatment-naive patients at early time points of infection (primary infection, PHI, and 6 months post-PHI) from the ANRS CO6 cohort, in the chronic phase of infection (viremic patients (VIR)) from the ANRS COPANA cohort, and during controlled infection, either cART-treated patients (cART) from the COPANA cohort and HIV controllers (HIC) from the ANRS CODEX cohort. (**D, E and F)** Evolution of sDPP4 levels before and during cART in 40 patients from the ANRS COPANA cohort. The values from the same patient are connected through a line to show the individual evolution of the sDPP4 levels before and after cART initiation. **(D)** Pre- and post-ART sDPP4 activity (UI/ml) in the 40 cART-treated patients. Twenty of these patients had received Protease inhibitors (PI). **(E)** Pre- and post-ART sDPP4 activity (UI/ml) in 20 NRTI-based cART-treated patients. **(F)** Pre- and post-ART sDPP4 activity (UI/ml) in the 20 patients on cART with PI-containing regimen. Pre-inf.= before HIV-1 infection; PHI= primary HIV-1 infection; M3 = 3 months post-seroconversion; M6 = 6 months post-seroconversion (panel A-B in the ACS) or post-PHI (panel C in the PRIMO cohort). For graphs A, B and C, the median + interquartile range are shown. For graphs A and C, the student t-test was used. For graphs B, D, E and F, the Wilcoxon sign-rank test for paired data was used. *: *p*< 0.05; **: *p*< 0.01; ***: *p*<0.001; ****: *p*<0.0001.

**Figure 2. Soluble blood DPP4 levels with regard to disease progression profiles.** Soluble DPP4 levels were quantified during primary HIV-1 infection in patients from the ANRS PRIMO cohort displaying distinct profiles of disease progression (Slow progressors, SP; Normal progressors, P; Rapid progressors, RP). HD= healthy donors. **(A)** sDPP4 enzymatic activity per ml of blood. The median levels were 18.0 for HD, 4.4 for SP and P, and 3.8 for RP. **(B)** Kaplan-Meier survival analysis of AIDS-related death by sDPP4 levels measured 6 months after seroconversion (M6) (≤ or > to the median) in treatment-naïve patients from the Amsterdam cohort. The dotted line corresponds to sDPP4 levels > median and the solid line to sDDP4 levels < median at M6. For graph A the Wilcoxon non-parametric test was used and for graph B the logrank test. The median and interquartile range is shown in graph A. **: *p*< 0.01; ***: *p*<0.001; ****: *p*<0.0001.

**Figure 3. *DPP4* mRNA levels in the gut in pathogenic and non-pathogenic SIV infection.** (**A, C, E)** Four intestinal compartments (ileum, jejunum, colon, rectum) from 5 rhesus macaques and 5 AGM were studied at an early phase of infection (day 65 pi.). CD4+ leukocytes were enriched from the distinct sections of the intestine. The values for the small intestine (ileum, jejunum) and large intestine (rectum, colon) were pooled. For MAC, the available material was: jejunum, colon and rectum for 5 animals, ileum for 3-5 animals; For AGM the available samples were: jejunum, colon and rectum for 4-5 animals, ileum for 2-5 animals). When the material was limited, we privileged the analyses of DPP4mRNA. **(A)** *DPP4* mRNA levels in intestinal CD4^+^ cells. **(B)** *DPP4* mRNA expression in CD4^+^ cells from the small intestine (jejunum) from the 5 MAC and 5 AGM plotted against plasma sDPP4 activity in blood from the same animals. **(C)** *RORC* mRNA levels in intestinal CD4^+^ cells. **(D**) *RORC* mRNA expression levels plotted against *DPP4* mRNA in CD4^+^ cells from the small intestine. **(E)** *TBX21* (*t-bet*) mRNA levels in intestinal CD4^+^ cells. **(F)** *TBX21* mRNA expression levels plotted against *DPP4* mRNA in CD4^+^ cells from the small intestine. The correlations were statistically significant when the two species were pooled, but not within individual species. **(B, D, F**) Each circle represents the value of one tissue sample. Black (full circles): MAC; white (open circles): AGM. For graphs A, C an E the Wilcoxon non-parametric test was used; for graphs B, D, and F the Spearman non-parametric correlation was used. The median and interquartile range are shown in graphs A, C and E. FC = fold change. *: *p*< 0.05; **: *p*< 0.01; ***: *p*<0.001.

**Figure 4. sDPP4 dynamics in blood after IL-21 immunotherapy of SIV-infected macaques. (A)** sDPP4 activity in blood was measured in a longitudinal analysis before initiation of IL-21 therapy (week 2 p.i.), at the end of IL-21 treatment (week 6 p.i.) and after IL-21 treatment (Weeks 10, 18 and 23 p.i.). The ratio of pre- and post-treatment sDPP4 levels are shown. **(B and C)** IL-17^+^ and IL-17^+^IFN-γ+ cells were measured in rectal biopsies on week 4 and week 6. **B)** Correlation between the percentage of intestinal IL-17^+^IFN-γ+ cells and the plasma sDPP4 activity (fold change from week 2 to week 10) **(C)** Correlation between the percentage of intestinal IL-17^+^ cells and plasma sDPP4 activity plasma sDPP4 activity (fold change from week 2 to week 10). **(D-F)** Correlation between sDPP4 activity in blood (fold change between week 2 and 10) and levels of **(D)** Ki67^+^CD4^+^ T cells in gut **(E)** blood IP-10 levels **(F)** blood sCD14 and **(G)** blood LPS after IL-21 therapy cessation at week 23 p.i. For graph A the Wilcoxon non-parametric test was used; for graphs B to G the Spearman non-parametric correlation. The median and interquartile range are shown in graph A. *: *p*<0.05; **: *p*< 0.01. Six rhesus macaques infected with SIVmac and treated with IL-21 and 6 rhesus control macaques infected with SIVmac were analyzed. CTRL: control monkeys; +IL-21: IL-21 treated monkeys.

| **Supplementary Table 1. Characteristics of the patients from the four distinct cohorts.** | | | | |
| --- | --- | --- | --- | --- |
| ***Cohort name*** | **ACS (n=136)** | **PRIMO (n=126)** | **COPANA (n=121)** | **CODEX (n=82) cART (n=71)** |
| **Male, n (%)** | 118 (86.8) | 109 (86.5) | 93 (76.9) | 43 (52.4) 49 (73.1) |
| **Age (years, median, IQR)** | 35 (30 – 40) | 35 (29 – 42) | 35 (30 – 42) | 46 (40 - 52) 42 (30-48) |
| **CD4^1^ pre-infection*, N (%)**  **Median (IQR)** | 76 (55.9)  895 (715-1205) | - | - | - - |
| **CD4^1^ at seroconversion**, N (%)**  **Median (IQR)** | 70 (51.5)  835 (620 –1160) | - | - | - - |
| **CD4 at PHI, N (%)**  **Median (IQR)** | 16 (11.8)  620 (435 – 880) | 125 (99.2)  554 (481 – 742) | - | - - |
| **CD4^1^ in chronic phase, N (%)**  **Median (IQR)** | - | - | 121 (100)  364 (286 – 543) | 82 (100) 58  651.5 (462 – 875) 568 (439-729) |
| **VL at seroconversion**, log copies/mL**  **N (%)**  **Median (IQR)** | 106 (77.9)  4.70 (3.76 – 5.26) | - | - | - - |
| **VL at PHI, log copies/mL**  **N (%)**  **Median (IQR)** | 35 (25.7)  5.50 (4.17 – 6.53) | 126 (100)  4.91 (4.28 – 5.52) | - | - - |
| **VL in chronic phase^2^,**  **log copies/mL**  **Median (IQR)** | - | - | 4.33 (3.86 – 4.81) | 1.30 (1.30 – 2.10) < 50 |
| For patients from the ACS, sera were available as follows: before infection (N=87), at primary HIV infection (PHI, N=36), at 3 months (M3, N=126) and 6 months post-seroconversion (M6, N=104). All 126 patients from the ANRS PRIMO cohort were studied at PHI and 35 of them were also studied at M6 post-PHI. The patients from the ANRS PRIMO cohort were divided into 33 rapid progressors (RP), 72 normal progressors (P) and 28 slow progressors (SP). The samples of the ANRS COPANA cohort were collected from 121 viremic (non-treated) patients. Among these 121 patients, 40 were followed longitudinally before and after cART initiation.  *VL: viral load; PHI: primary HIV infection; IQR: interquartile range; *closest measurement pre-infection to date of SC, within the interval -24 months/-3 months before SC, **Seroconversion as defined by the ACS;* ^1^ cells/mm^3^ ; ^2^ at time of sDPP4 measurement. | | | | |

**Supplementary Table 2. Comparison of the levels of**

**sDDP4 in HIV controllers (HIC) compared to those in**

**healthy donors (HD) and other HIV-infected individuals.**

**
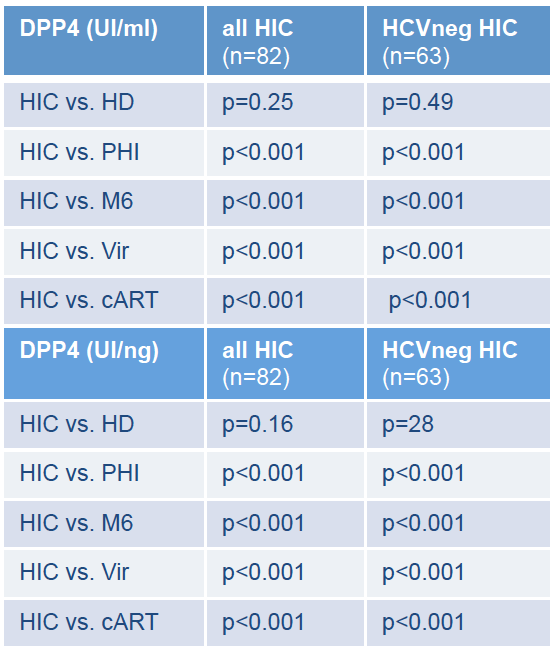
**

PHI: Patients in primary HIV infection; M6: people living

with HIV at six months post-infection, Vir= viremic patients,

cART: people living with HIV under cART treatment.

**SUPPLEMENTARY FIGURE LEGENDS**

**Supplementary Figure 1. Sequences of the PCR amplicons for three species (human, AGM, MAC).** The Taqman probes for (A) DPP4, (B) TBX21, (C) RORC and (D) 18S were designed by the manufacturer for human sequences. The corresponding sequences in the AGM and MAC species were identified according to the information provided by the manufacturer, which is approximate. Sequences covering the whole amplicons are depicted in the figure. The exact size of the PCR amplicons generated by the Taqman assays are 90, 62 and 62bp for DPP4, TBX1 and RORC, respectively. The exact location within the sequences shown here is unknown. The probes are 15-18bp long and located inside the yellow part. The nucleotides in red within each sequence correspond to the mid-position of the sequence containing the probe. The nucleotides highlighted in blue designate the nucleotides that are different between monkey and human sequences.

**Supplementary Figure 2. (**A). Comparison of enzymatic activity of sDPP4 in plasma compared to the absolute concentration of sDDP4 quantified in the same samples. The analyses were performed in samples from healthy donors. sDPP4 enzymatic activity and concentrations also correlated in plasma samples from HIV-infected patients (R>0.68, p<0.0001, not shown). **(B). Longitudinal evolution of soluble DPP4 activity levels from pre-infection until 6 monts post seroconversion.** Individual evolution over time in 11 patients from the Amsterdam Cohort, the same patients as shown in Figure 1B. The level at the distinct time points post infection was compared to the respective levels pre-infection using a non-parametric test for paired data. **(C-E) sDDP4 activity levels in cART treated patients described in Figure 2.** The median + interquartile range are shown.

**Supplementary Figure 3. Soluble DPP4 activity and concentration in blood during primary HIV-1 infection in patients with distinct disease progression profiles. (A)** Enzymatic activity. The median levels were respectively 18.0 for HD, 4.4 for SP and P, and 3.8 for RP. **(B)** Concentration of sDDP4. **(C)** Enzymatic activity normalized with respect to the protein concentration in blood. The median levels were respectively 0.037 for HD, 0.006 for SP and P, and 0.005 for RP. HD=healthy donors; SP=Slow progressors; P=Progressors; RP=Rapid progressors. Patients from the ANRS CO6 PRIMO cohort [48]. The Wilcoxon non-parametric test was used. The median and interquartile range are shown. **:*p*< 0.01; ***:*p*<0.001; ****: *p*<0.0001.

**Supplementary Figure 4. Levels of *DPP4* and *RORC* mRNA in CD4+ and CD4- cells of the gut.** CD4+ cells from four distinct gut sections were isolated from leukocytes of 5 Rhesus Macaques (MAC) and 5 African Green Monkeys (AGM) infected by SIV (65 days p.i.). The *DPP4* **(A)** and *RORC* **(B)** mRNA expression levels were quantified in the CD4+ cells and compared to the expression levels in the remaining leukocyte fraction. For MAC, enough material for the analyses were available for: jejunum from 5 animals, colon and rectum from 2-5 animals, ileum from 3 animals; AGM: jejunum, colon and rectum from 4-5 animals, ileum from 2 animals. When the material was limited, we privileged the analysis of the CD4+ cells as compared to the CD4^+^-depleted fraction. *DPP4* was expressed at higher levels in CD4+ cells. The same statistical analyses as described in Figure 3 were applied. MAC: closed circles, AGM: open circles. The median and interquartile range are shown.

*:*p*< 0.05; **:*p*< 0.01.
